# Transcriptional changes associated with resistance to inhibitors of epidermal growth factor receptor revealed using metaanalysis

**DOI:** 10.1186/s12885-015-1337-3

**Published:** 2015-05-07

**Authors:** Sidra Younis, Qamar Javed, Miroslav Blumenberg

**Affiliations:** 1 The R.O.Perelman Department of Dermatology, New York, USA; 2 Department of Biochemistry and Molecular Pharmacology, New York, USA; 3 NYU Cancer Institute, NYU Langone Medical Center, New York, USA; 4 Department of Biochemistry, Quaid-i-Azam University, Islamabad, 45320 Pakistan; 5 NYU School of Medicine, 455 First Avenue, New York, 10016 USA

**Keywords:** Development of resistance, Erlotinib, Gefitinib, Metabolic changes, Targeted therapy

## Abstract

**Background:**

EGFR is important in maintaining metabolic homeostasis in healthy cells, but in tumors it activates downstream signaling pathways, causing proliferation, angiogenesis, invasion and metastasis. Consequently, EGFR is targeted in cancers using reversible, irreversible or antibody inhibitors. Unfortunately, tumors develop inhibitor resistance by mutations or overexpressing EGFR, or its ligand, or activating secondary, EGFR-independent pathways.

**Methods:**

Here we present a global metaanalysis comparing transcriptional profiles from matched pairs of EGFR inhibitor-sensitive *vs.* -resistant cell lines, using 15 datasets comprising 274 microarrays. We also analyzed separately pairs of cell lines derived using reversible, irreversible or antibody inhibitors.

**Results:**

The metaanalysis identifies commonalities in cell lines resistant to EGFR inhibitors: in sensitive cell lines, the ontological categories involving the ErbB receptors pathways, cell adhesion and lipid metabolism are overexpressed; however, resistance to EGFR inhibitors is associated with overexpression of genes for ErbB receptors-independent oncogenic pathways, regulation of cell motility, energy metabolism, immunity especially inflammatory cytokines biosynthesis, cell cycle and responses to exogenous and endogenous stimuli. Specifically in Gefitinib-resistant cell lines, the immunity-associated genes are overexpressed, whereas in Erlotinib-resistant ones so are the mitochondrial genes and processes. Unexpectedly, lines selected using EGFR-targeting antibodies overexpress different gene ontologies from ones selected using kinase inhibitors. Specifically, they have reduced expression of genes for proliferation, chemotaxis, immunity and angiogenesis.

**Conclusions:**

This metaanalysis suggests that ‘combination therapies’ can improve cancer treatment outcomes. Potentially, use of mitochondrial blockers with Erlotinib, immunity blockers with Gefitinib, tyrosine kinase inhibitors with antibody inhibitors, may have better chance of avoiding development of resistance.

**Electronic supplementary material:**

The online version of this article (doi:10.1186/s12885-015-1337-3) contains supplementary material, which is available to authorized users.

## Background

Cancer is principally caused by changes in three types of genes i.e. oncogenes, tumor suppressor genes and DNA stability genes; common environmental factors contributing to these changes could be smoking, oncogenic viruses, occupational and environmental carcinogens and predisposing genetic polymorphisms [1,2]. Massive research efforts are ongoing to find treatments for cancer, still an unresolved problem and of course a heavy burden on health care. Targeting specific pathways and modulating the immune system are key strategies to control cancer progression and increase effectiveness of treatment [3].

EGFR, important growth factor receptor implicated in many cancers, is one of the targets for chemotherapy. EGFR belongs to ErbB family of tyrosine kinase receptors [4]. In tumors these receptors are activated by increased expression or structural rearrangement of receptor gene, mutation in ligand binding or tyrosine kinase domain or by the production of autocrine and paracrine ligands. On activation, EGFR dimerizes and triggers Ras-RAF-MEK-ERK-MAPK, JAK-STAT and other signaling cascades [4,5]. These pathways activate transcription factors, ultimately resulting in activation of cellular processes including proliferation, carbohydrate utilization, protein synthesis, angiogenesis, cell growth and cell survival [4,6]. In cancer cells, EGFR activation is important in maintaining the metabolic homeostasis and stimulates proliferation, invasion, angiogenesis, survival, decreased apoptosis, migration, differentiation and adhesion. Because of its central signaling position, EGFR is targeted in number of malignancies e.g. lung, colorectal, pancreatic, head and neck cancers, glioblastomas etc. [7].

To treat malignancies, EGFR activity is targeted with reversible, irreversible or antibody inhibitors and their combinations. Reversible inhibitors, e.g., Gefitinib and Erlotinib, compete for the intracellular ATP binding site of the kinase; the irreversible inhibitors, e.g., PF299804 and WZ4002, block the ATP binding site by covalent interaction with Cys773 in EGFR [8]. Antibody inhibitors block the extracellular ligand binding domain of EGFR thereby preventing ligand binding and receptor dimerization. Different types of inhibitors generate different transcriptional responses in EGFR-targeted cells [9].

Unfortunately, after a period, tumors inexorably develop resistance to inhibitors by e.g., overexpression of EGFR ligand, activating mutations in the tyrosine kinase or the ligand-binding domain, or by mutations in downstream or parallel signaling pathways, e.g., Axl or IGFIR [10-14]. Multiple studies focused on defining the secondary mutations that cause resistance to EGFR inhibitors [13-15]. However, much less attention has been paid to the transcriptional and metabolic changes that distinguish the resistant cells from the sensitive ones [12]. Therefore, we decided to explore the fundamental functional changes that distinguish the resistant cells from the sensitive ones, using transcriptional profiling. To cast a wide net, we used metaanalysis approach to find the differential gene expression between matched pairs of cell lines sensitive and resistant to EGFR inhibitors. Here we included eight studies with 15 distinct datasets directly comparing the transcriptional profiles in EGFR inhibitor-sensitive *vs.* resistant cell lines. The cell lines included non-small cell lung cancer, head and neck cancer, and epidermoid carcinoma cell lines. The inhibitors included both reversible and irreversible kinase inhibitors, as well as antibodies.

We found that in EGFR inhibitor-sensitive cell lines characteristically overexpressed gene ontologies are adhesion, negative regulation of cell proliferation, lipid metabolism and oncogenic processes involving ErbB receptors. But when cells become resistant, ontological categories associated with energy metabolism, immunity involving overexpressing inflammatory cytokines, responses to external and internal stimuli, proliferation and ErbB-independent oncogenic pathways are overexpressed. The specific resistance to Gefitinib apparently develops by overexpressing immunomodulatory genes; resistance to Erlotinib by energy producing mitochondrial pathways; resistance to irreversible inhibitors by overexpressing EGFR ligands, whereas resistance to antibody inhibitors develops differently from the resistance to tyrosine kinase inhibitors.

## Methods

### Downloading the data files

The overall flowchart of our methodology is graphically represented in Additional file 1: Figure S1.

Different microarray platforms used for transcriptional profiling produced different, characteristic data files, which were worked up separately and then synchronized. The CEL or TXT files deposited in these studies were first downloaded and unzipped. For each study, data obtained from sensitive and resistant cell lines were saved in different columns of excel spread sheets. Datasets obtained from Affymetrix studies were combined and analyzed using RMAExpress for quality control [16,17]. For non-Affymetrix studies, where we could not run RMAExpress quality control, we downloaded already normalized, _RAW.tar files and used these without further modifications, as submitted by the original authors.

### Grouping studies for analysis using RankProd software

RankProd package analyses gene expression microarray data specifically to identify differentially expressed genes. RankProd uses non-parametric rank product method to detect genes that are consistently found among the most strongly upregulated ones and the most strongly downregulated ones in a number of replicate experiments, comparing two different condition [18]. We have combined into a single spreadsheet microarray data for sensitive and resistant cell lines with 20552 common genes in all datasets using data-loader [17]. Five datasets comprising 214 microarrays and 28235 genes for Gefitinib-sensitive and resistant cell lines were combined into a single excel spreadsheet and analyzed using RankProd. Differentially expressed genes in each of the class were recorded. Microarray data for the seven datasets comprising forty Erlotinib-sensitive and resistant microarrays, having 32062 common genes were combined for analysis using RankProd software [17].

We have pooled and compared the microarray data for EGFR irreversible inhibitors from two datasets, fourteen microarrays and 21631 common genes. For studying EGFR antibody inhibitors responses we found a single study with 3 microarrays from Cetuximab-sensitive and 3 from resistant cell lines, with 48607 genes.

We used the RankProd Software to find out the genes differentially expressed in EGFR inhibitor-sensitive and resistant cell lines with p-values better than 10^−4^. For each analysis we derived two tables, one representing the ontological categories over expressed in sensitive cell lines and second table with gene ontologies overregulated in resistant cell lines [18]. The results of the RankProd analysis are presented in Additional file 2: Figure S2.

### DAVID analysis

We used online Database for Annotation, Visualization and Integrated Discovery (DAVID) software as described before [17,19]. We also generated clusters, which reduced overlaps and redundancies in regulated ontological categories, for Erlotinib-, irreversible inhibitor- and Cetuximab-sensitive and resistant cell lines. These are provided in Additional files 3,4,5,6,7, and 8.

## Results

### Datasets characterization

Inhibitors have been studied principally to find out their mechanism of action in different cancer types. We here aim to study the differential gene expression in EGFR inhibitor-sensitive *vs.* resistant cancer cell lines. We searched GEO Datasets using key term “EGFR Resistance” and, limiting our choices to human cell lines, selected studies that directly compare transcriptional profiles of matched EGFR inhibitor-sensitive *vs.* resistant datasets (Table 1). We found 8 appropriate studies comprising 15 datasets and 274 microarrays. In seven datasets EGFR inhibitor-sensitive *vs.* resistant cell lines were compared without any other treatment. In one study comprising four datasets (GSE38310), two different types of Erlotinib-resistant cell lines were originated from Erlotinib-sensitive cell lines and then treated either with DMSO or Erlotinib. We compared DMSO-treated sensitive cell lines with DMSO-treated resistant cell lines and Erlotinib-treated sensitive cell lines with Erlotinib-treated resistant cell lines. In a Gefitinib study comprising three datasets [10], sensitive and resistant cell lines were treated with Gefitinib and EGF separately or in combination (GSE34228). In one study Erlotinib-sensitive and resistant cell lines were treated with miR-7 (GSE40130). In another study, GSE38404, resistant cell lines were produced by exposing the sensitive cell lines to inhibitor for different time periods. We have combined data from all the resistant cell lines for metaanalysis.

**Table 1:** **EGFR inhibitors-sensitive versus resistant cell lines datasets used in metaanalysis of microarrays**

Sr. No	Acc. No.	Platform	Set	S+R	S cell lines	R Cell lines	Cell Type	Pretreatment	Inhibitor
1	GSE34228	Agilent-014850	1	26+26	PC9	PC9GR	Non-Small Cell Lung Cancer	No	Gefitinib
			2	26+26	PC9	PC9GR	Non-Small Cell Lung Cancer	EGF	Gefitinib
			3	26+26	PC9	PC9GR	Non-Small Cell Lung Cancer	IRS	Gefitinib
			4	26+26	PC9	PC9GR	Non-Small Cell Lung Cancer	EGF+IRS	Gefitinib
2	GSE10696	Affy HG_ U133_Plus_2.0	1	3+3	A431	A431GR	A431 cancer cell line	No	Gefitinib
3	GSE38310	Illumina HumanHT-12 V3.0	1	3+3	HCC827	T15-2	Non-Small Cell Lung Cancer	DMSO	Erlotinib
			2	3+3	HCC827	T15-3	Non-Small Cell Lung Cancer	Erlotinib	Erlotinib
			3	3+3	HCC827	ER-3	Non-Small Cell Lung Cancer	DMSO	Erlotinib
			4	3+3	HCC827	ER-3	Non-Small Cell Lung Cancer	Erlotinib	Erlotinib
4	GSE40130	Illumina HumanWG-6 v3.0	1	2+3	HN5	FADU	Head and Neck cancer cell lines	No	Erlotinib
		Illumina HumanHT-12 V4.0	2	2+3	HN5	FADU	Head and Neck cancer cell lines	miR-7	Erlotinib
5	GSE49135	Illumina HumanHT-12 V4.0	1	3+3	HN5	HN5ER	Head and Neck cancer cell lines	No	Erlotinib
6	GSE37699	Affy_HG_U133A_2	1	3+3	NSCLC	NCl-H1975	Non-Small Cell Lung Cancer	No	WZ4002
7	GSE38404	Affy_HG_U133A_2	1	2+6	NSCLC	PFR31, PFR32	Non-Small Cell Lung Cancer	No	PF299804
8	GSE21483	Affy HG_ U133_Plus_2.0	1	3+3	SCC1	1Cc8	Skin cancer cell line	No	Cetuximab
	Total		15	274					

Illumina microarrays were used in majority of datasets, comprising 3 studies and 7 datasets. In four studies, comparing four datasets Affymetrix microarrays were used. Agilent microarrays were used in one study comparing four datasets. Different studies used different types of cell lines for example Non-Small Cell Lung Cancer, Head and Neck Cancer, A431 and Skin cancer cell lines; therefore, we have also individually compared datasets from individual studies (data not shown).

Information about the type of sensitive and resistant cells, pretreatment and type of EGFR inhibitor, labeling accession number, platform, number of datasets, number of sensitive and resistant microarray chips in each dataset is summarized in Table 1.

We have compared these datasets using different approaches; 1) datasets comparing all EGFR inhibitor-sensitive *vs.* resistant cell lines; 2) Gefitinib- and 3) Erlotinib-sensitive *vs.* resistant cell lines; 4) irreversible inhibitor-sensitive *vs.* resistant cell lines; 5) Cetuximab-sensitive *vs.* resistant cell lines [11]. We used RankProd software to select differentially expressed genes in these individual groups i.e. sensitive *vs.* resistant, with results graphically presented in (Additional file 2: Figure S2).

Ontological categories overrepresented in lists of differentially expressed genes were identified using DAVID [19]; complete lists are given in Additional files 3, 4, 5, 6 and 7. To condense redundancies, we have also identified clusters for differentially expressed ontological categories in the sensitive *vs.* Erlotinib-, Irreversible- and Cetuximab-resistant groups (see below).

### Global comparison of all EGFR inhibitors-sensitive *vs.* resistant cell lines

We have compared overexpressed ontological categories in all sensitive *vs*. resistant cell lines. Table 2a contains the 10 categories with the best p-values; in Table 2b, we selected the characteristically different categories between sensitive and resistant cells with p-values better than 10^−4^.

**Table 2:** **Global comparison of the ontological categories differentially expressed in EGFR inhibitors-sensitive** ***vs.*** **resistant cell lines**

	All studies: Overexpressed in sensitive cells			Overexpressed in resistant cells	
	Term	p-value		Term	p-value
**Table 2a**					
**O**	Pathways in cancer	4.9E-15	**T**	positive regulation of biosynthetic process	3.6E-17
**MM**	plasma membrane part	7.7E-12	**T**	positive regulation of cellular biosynthetic process	5.2E-17
**CC**	regulation of cell proliferation	1.3E-10	**T**	positive regulation of macromolecule Metabolic process	2.3E-16
**MM**	endomembrane system	1.4E-10	**R**	response to endogenous stimulus	8.9E-16
**MM**	cell fraction	2.1E-09	**T**	positive regulation of nitrogen compound Metabolic process	1.6E-15
**A**	regulation of cell death	2.2E-09	**T**	positive regulation of macromolecule biosynthetic process	9.3E-15
**A**	regulation of programmed cell death	2.6E-09	**CC**	regulation of cell proliferation	4.9E-14
**Mt**	positive regulation of macromolecule Metabolic process	2.6E-09	**R**	response to hormone stimulus	6.9E-14
**CC**	cell proliferation	5.7E-09	**R**	response to organic substance	9.6E-14
**T**	protein complex biogenesis	7.0E-09	**CC**	positive regulation of nucleobase, nucleoside, nucleotide and nucleic acid Metabolic process	1.2E-13
**Table 2b**					
**A**	negative regulation of cell death	1.4E-07	**A**	regulation of apoptosis	3.3E-10
**AD**	cell migration	5.0E-08	**A**	negative regulation of apoptosis	4.1E-10
**AD**	cell adhesion	2.3E-07	**A**	anti-apoptosis	8.1E-08
**AD**	localization of cell	1.2E-06	**CC**	cell cycle	8.2E-06
**AD**	cell projection	3.2E-06	**CC**	regulation of cell size	3.4E-05
**AD**	Focal adhesion	4.3E-05	**CY**	cytoskeletal protein binding	3.2E-06
**CC**	negative regulation of cell proliferation	9.0E-05	**CY**	actin cytoskeleton	1.0E-04
**CC**	regulation of DNA Metabolic process	1.5E-04	**DF**	negative regulation of cell differentiation	6.1E-06
**CC**	mitotic cell cycle	5.9E-04	**E**	regulation of oxidoreductase activity	5.1E-05
**CC**	regulation of cell size	6.4E-04	**E**	positive regulation of oxidoreductase activity	4.2E-04
**IM**	response to wounding	4.4E-05	**IM**	immune system development	1.8E-09
**IM**	immune system development	6.2E-04	**IM**	response to wounding	2.4E-08
**IM**	T cell activation	6.8E-04	**IM**	regulation of cytokine production	1.1E-07
**IM**	humoral immune response	8.4E-04	**IM**	positive regulation of immune system process	4.3E-06
**M_c**	oligosaccharide Metabolic process	7.0E-04	**IM**	positive regulation of cell activation	2.6E-05
**M_l**	regulation of lipid Metabolic process	6.8E-06	**IM**	inflammatory response	2.8E-05
**M_l**	phosphoinositide Metabolic process	7.3E-05	**IM**	wound healing	1.6E-04
**M_l**	positive regulation of lipid Metabolic process	1.0E-04	**IM**	immune response	1.8E-04
**M_l**	lipid biosynthetic process	1.1E-04	**IM**	defense response	4.5E-04
**M_l**	phospholipid Metabolic process	2.3E-04	**IM**	regulation of production of molecular mediator of immune response	9.1E-04
**M_l**	cellular lipid catabolic process	4.2E-04	**M_c**	monosaccharide Metabolic process	4.3E-06
**M_l**	glycerolipid Metabolic process	5.4E-04	**M_c**	hexose Metabolic process	1.2E-05
**M_l**	glycerophospholipid Metabolic process	7.0E-04	**M_c**	glucose Metabolic process	5.8E-05
**M_l**	lipoprotein particle clearance	9.2E-04	**M_c**	regulation of cellular ketone Metabolic process	2.0E-04
**O**	Oncogenesis	3.4E-05	**M_l**	regulation of lipid Metabolic process	2.6E-07
**O**	ErbB signaling pathway	4.2E-04	**M_l**	regulation of lipid biosynthetic process	1.1E-04
**O**	Wnt signaling pathway	7.7E-04	**M_l**	regulation of fatty acid Metabolic process	4.2E-04
			**M_l**	positive regulation of lipid Metabolic process	4.9E-04
**Legend:**			**MO**	regulation of cell motion	2.8E-12
**A**	Apoptosis		**O**	Pathways in cancer	2.8E-10
**AD**	Adhesion		**O**	regulation of DNA Metabolic process	1.2E-04
**CC**	Cell cycle		**O**	Ras protein signal transduction	2.1E-04
**CY**	Cytoskeleton		**O**	Oncogenesis	4.0E-04
**DF**	Differentiation		**R**	response to steroid hormone stimulus	6.2E-12
**E**	Energy		**R**	response to insulin stimulus	1.5E-07
**Mt**	Metabolism		**R**	response to vitamin	1.2E-06
**IM**	Immunity		**R**	response to lipopolysaccharide	1.8E-06
**M_c**	Metabolism-carbohydrates		**R**	response to glucocorticoid stimulus	2.6E-06
**M_l**	Metabolism-lipids		**R**	response to hydrogen peroxide	1.4E-05
**MM**	Membrane		**R**	cellular response to stress	3.1E-05
**MO**	Motility		**V**	regulation of angiogenesis	4.6E-06
**O**	Oncogenesis		**V**	myeloid leukocyte activation	4.4E-05
**R**	Response to stimuli		**V**	positive regulation of angiogenesis	4.2E-04
**T**	Transcription/translation				
**V**	Vasculogenesis				
**Sensitive**			**Resistant**		
**Table 2c**					
**ErbB Signalling pathway**			**Ras protein signal transduction**		
BCL2-associated agonist of cell death			v-ral oncogene homolog A		
mitogen-activated protein kinase 9			FERM, RhoGEF and pleckstrin domain		
phosphoinositide-3-kinase beta			nischarin		
glycogen synthase kinase 3 beta			IGF1 (somatomedin C)		
c-abl oncogene 1			Rho GTPase activating protein 6		
v-crk sarcoma virus CT10			X-associated ankyrin-containing protein		
mitogen-activated protein kinase 8			ras homolog gene family, member A		
ribosomal protein S6 kinase			myosin IXB		
mitogen-activated protein kinase 10			mitogen-activated protein kinase 1		
phosphoinositide-3-kinase alpha			GRB2-related adaptor protein 2		
phospholipase C, gamma 1			Rho GTPase activating protein 5		
CDK inhibitor 1A (p21, Cip1)			soc-2 suppressor of clear homolog		
epiregulin			mitogen-activated protein kinase 14		
v-myc oncogene homolog			ropporin, rhophilin associated protein 1B		
phosphoinositide-3-kinase subunit 5			r-ras oncogene homolog		
v-erb-b2 oncogene homolog 2			CDC42 effector protein		
v-aktoncogene homolog 1			fibroblast growth factor 2 (basic)		
calcium/calmodulin-dep. protein kinase II			G protein alpha 12		
protein kinase C, alpha			parathyroid hormone		
neuregulin 1			GRB2-related adaptor protein		
NCK adaptor protein 1			muscle RAS oncogene homolog		
PTK2 protein tyrosine kinase 2			WAS protein family, member 2		
phosphoinositide-3-kinase delta			SHC transforming protein 1		
betacellulin			MAPKAP 2		
ribosomal protein S6 kinase 2			Rho guanine nucleotide exchange factor 3		
jun oncogene			ATP-binding cassette, member 1		
p21 (Cdc42/Rac)-activated kinase 2			cofilin 1 (non-muscle)		
translation initiation factor 4E binding			CDC42 effector protein 4		
phospholipase C, gamma 2			neurofibromin 1		
			neurotrophic tyrosine kinase, type 1		
			ral GDF stimulator-like 2		
			linker for activation of T cells		
**Sensitive**			**Resistant**		
**Table 2d**					
**Complement System**					
complement component 2			complement component 1, q A chain		
complement component 3a receptor 1			complement component 1, q B chain		
complement component 7			complement factor H		
complement component 8, alpha					
complement component 8, beta					
complement component 9					
complement factor B					
complement factor H					
complement factor I					
**Chemokines**					
chemokine (C-C motif) ligand 2			chemokine (C-C motif) ligand 13		
chemokine (C-C motif) ligand 22			chemokine (C-C motif) ligand 19		
chemokine (C-C motif) ligand 23			chemokine (C-C motif) ligand 2		
chemokine (C-C motif) ligand 24			chemokine (C-C motif) ligand 20		
chemokine (C-C motif) receptor 5			chemokine (C-C motif) ligand 8		
			chemokine (C-C motif) receptor 5		
			chemokine (C-X-C motif) ligand 1		
			chemokine (C-X-C motif) ligand 13		
			chemokine (C-X-C motif) ligand 6		
**Interleukins**					
interleukin 1, alpha			interleukin 1 family, member 6		
interleukin 10			interleukin 1, alpha		
interleukin 2 receptor, alpha			interleukin 1, beta		
interleukin 6 (interferon, beta 2)			interleukin 10		
interleukin 8			interleukin 10 receptor, beta		
			interleukin 13		
			interleukin 22		
			interleukin 5		
			interleukin 6 (interferon, beta 2)		
			interleukin 9		

The data represent the ontological and functional categories identified as overrepresented in the lists of differentially expressed genes, when compared to all genes in the human genome. The lists are provided by the DAVID analysis program [17,19]. a) Top ten categories with best p-values. b) Selected categories with p-value better than 10^-4^. For complete list of categories with p-values better than 10^-4^ see Additional file 3; the selected categories represent our choices as the ones that illustrate the best differences between resistant and sensitive cell lines. c) Genes expressed in ErbB signaling pathway and Ras protein signal transduction ontologies of sensitive and resistant cell lines respectively. d) Genes expressed in immune system development of sensitive versus resistant cell lines.

In Table 2a, we find that in the sensitive cell lines the genes related to systems involved in transport of materials across the membranes are overexpressed, whereas in the resistant cell lines the genes related to the metabolism and macromolecule biosynthesis, especially of proteins, are overexpressed. Although we have found genes associated with regulation of the protein translation in both groups, this category is more significant in the resistant cell lines. Both EGFR inhibitor-sensitive and resistant cell lines overexpress cell proliferation genes, however the sensitive cell lines tend to overexpress apoptosis and cancer-related genes as well. Interestingly, the resistant cell lines seem significantly more responsive to various endogenous and exogenous stimuli than the sensitive ones.

Oncogenesis-related genes and pathways are up-regulated in both sensitive and resistant cell lines. As reported in previous studies, in sensitive cell lines tumor growth seems dependent on the oncogene activation through ErbB receptor kinases [15]. In contrast, in resistant cell lines often the Ras pathway is activated independently of receptors (Table 2b). We note that while Ras is considered a downstream target of EGFR signaling in noncancerous cells [20], the EGFR- and Ras-associated genes comprise widely different groups (Table 2c).

Interestingly, we have observed that in the sensitive cell lines lipids are preferentially metabolized as the source of energy. But as cells become resistant, both carbohydrates and lipids are metabolized to provide energy. In addition, resistant cell lines are inducing the expression of energy generating genes, including the oxidoreductases. This observation suggests that energy production is an important matter for development of resistant cell lines.

Genes involved in responses to various extracellular and intracellular stimuli, for example steroid hormone, hydrogen peroxide and stress, are over expressed significantly in the resistant cell lines compared to sensitive ones. These responses may be related either to cells’ protection from toxins or overall cell survival, as stress and hydrogen peroxide responses prepare the cell for a toxic environment and steroids stimulate proliferation in certain cancers [21,22]. Those cancer cells that increase expression of genes responsive to these stimuli tend to adapt and survive [23].

We have also observed that certain processes related to immunity are overexpressed in the sensitive as well as in resistant cell lines, but we found that ontological categories for these processes have significantly better p-values in the resistant ones. Further, detailed study of the genes involved in immunity revealed that the innate immunity involving the complement component system is active in sensitive cells. In contrast, the resistant cells utilize both innate and adaptive immune systems, especially involving the cytokines and chemokines (Table 2d).

In EGFR inhibitor-sensitive cell lines, cell death and proliferation are relatively suppressed. But in resistant cell lines proliferation is relatively increased, while differentiation is decreased, thus favoring cancer cells growth and persistence. Another major distinction is that sensitive cell lines strongly express adhesion-related genes, whereas in resistant cells the genes related to cell movement are overexpressed. This suggests that the resistant cells have increased tendency to metastasize, perhaps *via* EMT [13,24].

### Comparison of Erlotinib-sensitive *vs.* resistant cell lines

Surprisingly, protein biosynthesis was the only ontological category in the top ten differentially expressed ones in Erlotinib-sensitive cell lines (Table 3a). In the resistant cells, we have also found mitochondria, immunity and cytoskeleton genes in the top ten ontological categories. In Erlotinib-sensitive cell lines, carbohydrate and protein metabolism genes are overexpressed with very good p-values, 10^−21^ (Table 3b). These lines seem to utilize the maximum of their energy reservoirs from glucose and protein molecules. Conversely, in the Erlotinib-resistant cell lines, glycolysis and gluconeogenesis are suppressed and, importantly, genes related to mitochondria and mitochondrial processes are remarkably boosted. These results suggest that increased production of energy to support cellular metabolic processes and survival are very important in the development of Erlotinib resistance. From these observations we suggest that energy level could be the limiting factor for the tumor cells survival in the presence of EGFR inhibitors.

**Table 3:** **Comparison of ontological categories differentially expressed in Erlotinib-sensitive** ***vs.*** **resistant cell lines, using DAVID program**

	Erlotinib: Overexpressed in sensitive cells			Overexpressed in resistant cells	
	Term	p_value		Term	p_value
**Table 3a**					
**T**	translation	5.6E-62		cytosol	3.0E-20
**T**	translational elongation	6.3E-45	**T**	translational elongation	3.1E-18
**T**	structural constituent of ribosome	1.0E-43	**T**	structural constituent of ribosome	3.4E-18
	cytosol	3.7E-41	**T**	ribosomal subunit	3.5E-17
**T**	ribosome	4.5E-41	**T**	cytosolic ribosome	2.4E-16
**T**	3' -UTR-mediated translational regulation	3.8E-40	**E**	mitochondrion	1.5E-15
**T**	ribonucleoprotein complex	5.0E-40	**T**	Ribosome	2.6E-14
**T**	Ribosome	4.6E-37	**T**	ribosome	2.6E-14
**T**	ribosomal subunit	9.9E-36	**IM**	Influenza Infection	4.2E-14
**T**	Protein biosynthesis	2.0E-33	**CY**	intracellular non-membrane-bounded organelle	8.2E-13
**Table 3b**					
**CC**	Cell cycle	1.8E-04	**CC**	mitotic cell cycle	3.2E-08
**E**	mitochondrion	2.4E-10	**CC**	M phase of mitotic cell cycle	9.1E-06
**E**	intramolecular oxidoreductase activity	4.4E-04	**CY**	actin cytoskeleton	5.3E-06
**M_c**	glycolysis	2.1E-07	**E**	mitochondrial inner membrane	7.1E-12
**M_c**	Metabolism of carbohydrates	3.3E-06	**E**	mitochondrial envelope	7.2E-12
**M_c**	carbohydrate catabolic process	7.5E-05	**E**	oxidative phosphorylation	1.5E-10
**M_c**	Glycolysis / Gluconeogenesis	1.7E-04	**E**	hydrogen ion transmembrane transporter activity	1.8E-10
**M_c**	glucose metabolic process	6.4E-04	**E**	generation of precursor metabolites and energy	3.4E-10
**T**	Metabolism of proteins	5.4E-29	**E**	Oxidoreductase	6.6E-10
**T**	ribosome biogenesis	6.6E-21	**E**	Dehydrogenase	3.9E-09
**T**	ncRNA metabolic process	4.7E-19	**E**	Integration of energy metabolism	1.6E-06
**T**	mitochondrial ribosome	4.0E-05	**E**	electron transport chain	1.8E-05
			**E**	mitochondrial lumen	1.9E-04
			**E**	mitochondrial matrix	1.9E-04
			**E**	NADH dehydrogenase (ubiquinone) activity	2.3E-04
			**E**	mitochondrial ATP synthesis coupled electron transport	2.8E-04
			**T**	Metabolism of proteins	2.7E-09
			**T**	Protein biosynthesis	1.6E-07
			**T**	Gene Expression	1.5E-06

a) Top ten categories with best p-values. b) Selected categories with p-value better than 10^-4^ (see Additional file 4).

### Comparison of Gefitinib-sensitive *vs.* resistant cell lines

In the Table 4a, we show that the cancer-related genes and pathways are preferentially activated in the sensitive cell lines. But in the cells resistant to Gefitinib, the ontological categories related to macromolecule biosynthesis, specifically of proteins, are most significantly overexpressed. Apparently, the sensitive cell lines have higher tendency for attachment, as the adhesion related genes are upregulated in these, whereas the resistant cell lines are significantly more responsive to endogenous and exogenous stimuli. In the sensitive cell lines ontological categories related to cell cycle, metabolism of proteins and signaling are also up regulated, but the p-values are not as significant as in the resistant cell lines. Tumor related pathways and cell cycle-related genes are also relatively upregulated in resistant cell lines.

**Table 4:** **Comparison of ontological categories differentially expressed in Gefitinib-sensitive** ***vs*** **. resistant cell lines**

	Gefitinib: Overexpressed in sensitive cells			Overexpressed in resistant cells	
	Term	p_value		Term	p_value
**Table 4a**					
**O**	Pathways in cancer	6.8E-14	**T**	positive regulation of biosynthetic process	1.7E-11
**O**	Pancreatic cancer	1.2E-07	**T**	positive regulation of macromolecule metabolic process	3.1E-11
**T**	protein complex assembly	3.7E-06	**T**	positive regulation of cellular biosynthetic process	3.5E-11
**T**	protein complex biogenesis	3.7E-06	**T**	positive regulation of nitrogen compound metabolic process	1.6E-10
**CC**	regulation of cell proliferation	3.9E-06	**T**	positive regulation of macromolecule biosynthetic process	4.4E-10
**O**	Bladder cancer	7.8E-06	**R**	response to endogenous stimulus	5.1E-10
**S**	regulation of protein kinase cascade	8.7E-06	**CC**	positive regulation of nucleobase, nucleoside, nucleotide and nucleic acid metabolic process	3.6E-09
**AD**	cell adhesion	9.0E-06	**R**	response to hormone stimulus	8.1E-09
**AD**	biological adhesion	9.8E-06	**O**	Pathways in cancer	1.0E-08
**T**	positive regulation of macromolecule metabolic process	1.0E-05	**T**	positive regulation of transcription	1.5E-08
**Table 4b**					
**A**	regulation of cell death	3.6E-05	**A**	negative regulation of programmed cell death	1.0E-06
**A**	negative regulation of cell death	4.4E-05	**CC**	regulation of cell proliferation	3.5E-08
**A**	regulation of apoptosis	7.9E-05	**CC**	positive regulation of cell proliferation	1.9E-06
**CC**	cell proliferation	1.6E-05	**CC**	cell cycle	2.7E-04
**M_l**	regulation of lipid metabolic process	3.1E-05	**CY**	cytoskeleton organization	5.3E-06
**M_l**	positive regulation of lipid metabolic process	3.4E-04	**CY**	cell morphogenesis	2.0E-04
**O**	ErbB signaling pathway	5.2E-04	**E**	regulation of oxidoreductase activity	4.9E-04
			**HY**	response to hypoxia	8.7E-06
			**HY**	response to reactive oxygen species	2.4E-04
			**HY**	response to hydrogen peroxide	6.3E-04
			**IM**	immune system development	4.1E-07
			**IM**	regulation of cytokine production	9.2E-07
			**IM**	regulation of cytokine biosynthetic process	2.8E-06
			**IM**	response to wounding	7.3E-05
			**IM**	positive regulation of cytokine biosynthetic process	1.1E-04
			**IM**	Signaling in Immune system	1.3E-04
			**M_c**	glucose metabolic process	5.1E-04
			**M_l**	regulation of lipid metabolic process	2.9E-04
			**R**	response to steroid hormone stimulus	4.5E-08
			**R**	response to nutrient levels	3.6E-07
			**R**	response to extracellular stimulus	6.8E-07
			**R**	response to drug	2.5E-06
			**R**	response to nutrient	3.7E-05
			**R**	response to vitamin	3.7E-05
			**R**	response to corticosteroid stimulus	4.3E-05
			**R**	response to glucocorticoid stimulus	9.1E-05
			**R**	response to lipopolysaccharide	1.7E-04
			**R**	response to abiotic stimulus	3.4E-04
			**R**	negative regulation of response to external stimulus	6.8E-04
			**V**	hemopoiesis	1.2E-07
			**V**	leukocyte differentiation	9.4E-06
			**V**	blood circulation	4.9E-05
			**V**	circulatory system process	4.9E-05
			**V**	lymphocyte differentiation	7.4E-05
			**V**	regulation of angiogenesis	1.1E-04
			**V**	blood vessel development	1.2E-04
			**V**	vasculature development	1.8E-04

a) Top ten categories with best p-values. b) Selected categories with p-value better than 10^-4^ (see Additional file 5).

Interestingly, we have not found any ontological category related to immune system development overexpressed in the sensitive cell lines. In contrast, in the resistant cell lines immunity systems, specifically biosynthesis of cytokines genes, are boosted (Table 4b). This suggests that Gefitinib-resistant cell lines use cytokines of the immune systems to develop resistance against Gefitinib and thus maintain tumor cells growth and progression [25].

In resistant cells the ontological categories related to responses to various stimuli and angiogenesis were robustly and consistently overexpressed. We have also found increased expression of genes responsive to reactive oxygen species, which ultimately tends to activate apoptosis and immunity pathways in these cells. Expression of genes related to hypoxia has also been seen in the resistant cell lines, which may increase their drug resistance and ultimately their growth rate [26].

Both in Gefitinib-sensitive and in resistant cell lines proliferation and apoptosis are regulated. However, in the resistant cell lines, the cell death processes are negatively regulated and the cell division is enhanced, suggesting that resistant cell lines have higher propensity to survive. Meanwhile, responses to various endogenous and exogenous stimuli are enhanced, maybe to cope with stresses from environment. In addition, immunity genes and cytokine biosynthesis are upregulated to help cancer cells to survive and grow.

Interestingly, we have observed that lipid metabolism genes are prominent in the sensitive cell lines, whereas oxidoreductases, as well as both glucose and lipid metabolism, i.e. genes related to production of energy, are more prominent in the resistant cell lines. Energy requirements for the tumor cells are met preferentially by formation of new blood vessels but also by glucose and lipid metabolism.

### Comparison of sensitive *vs.* resistant cell lines obtained using irreversible EGFR inhibitors

We have combined the microarray data from the cell lines selected as resistant to irreversible EGFR inhibitors WZ4002 and PF299804. Unexpectedly, in the top ten categories from the sensitive cell lines, genes related to membrane systems are found (Table 5a). In contrast, the extracellular region-related ontological categories are upregulated in the resistant cell lines. Cell cycle and lipid metabolism categories are relatively upregulated in the sensitive cell lines, similar to what was observed in reversible EGFR inhibitors-sensitive cell lines (Table 4b). Ontological categories related to cell division were found in both groups, however, in the resistant cell lines, cell division is positively regulated with better p-value than in sensitive cell lines. Importantly, the immunity genes are relatively overexpressed in resistant cell lines, reinforcing our observation that cytokine overexpression is one of the survival strategies gained by resistant cell lines (Table 5b).

**Table 5:** **Comparison of ontological categories differentially expressed in irreversible inhibitor-sensitive** ***vs*** **. resistant cell lines**

	Irreversible: Overexpression in sensitive cells			Overexpression in resistant cells	
	Term	p_value		Term	p_value
**Table 5a**					
**MM**	endomembrane system	3.8E-09	**EC**	extracellular region	1.6E-08
**MM**	Golgi apparatus	2.4E-07		ectoderm development	3.3E-07
**MM**	cell fraction	1.0E-05	**IM**	response to wounding	4.3E-07
**CC**	cell division	2.1E-05	**EC**	extracellular region part	1.3E-06
**MM**	organelle membrane	3.1E-05	**EC**	extracellular space	2.4E-06
**MM**	nuclear envelope-endoplasmic reticulum network	5.9E-05		epidermis development	8.4E-06
**R**	response to organic substance	8.1E-05	**CC**	regulation of cell proliferation	1.1E-05
**MM**	endoplasmic reticulum membrane	1.0E-04	**CC**	regulation of smooth muscle cell proliferation	6.5E-05
**CC**	Mitosis	1.1E-04	**IM**	wound healing	7.3E-05
**MM**	1p22.1	1.5E-04	**CC**	positive regulation of smooth muscle cell proliferation	1.9E-04
**Table 5b**					
**CC**	cell cycle	3.1E-04	**IM**	defense response	5.9E-04
**M_l**	steroid metabolic process	4.3E-04	**IM**	inflammatory response	9.2E-04
			**O**	epidermal growth factor receptor binding	2.5E-04
			**O**	ErbB signaling pathway	4.2E-04
**Genes overexpressed in resistant cells**					
**Table 5c**					
**EGFR Binding ligands**		**ErbB sig pathway**			
amphiregulin; amphiregulin B		Cas-Br-M ecotropic retroviral transforming sequence c			
epidermal growth factor receptor		amphiregulin; amphiregulin B			
Epiregulin		epidermal growth factor receptor			
heparin-binding EGF-like growth factor		epiregulin			
		heparin-binding EGF-like growth factor			
		transforming growth factor, alpha			
		v-erb-b2 oncogene homolog 3			

a) Top ten categories with best p-values. b) Selected categories with p-value better than 10^-4^ (see Additional file 6). c) Genes expressed in EGFR binding and ErbB signaling pathway ontologies in resistant cell lines.

Surprisingly, we found increased expression of EGFR ligands in cells selected using irreversible inhibitors (Table 5b). Perhaps these cells are still dependent on EGFR-mediated signaling cascades. This differs from observations in cell lines selected for resistance to reversible inhibitors, which use alternative pathways for oncogenes activation (Tables 2b and 3b).

### Comparison of Cetuximab-sensitive *vs.* resistant cell lines

For the analysis of cells selected as resistant to antibody inhibitor Cetuximab, we could find only a single study including six microarrays [27], which means that the statistical robustness of the results is reduced. Interestingly, we found that the differential gene expression in Cetuximab-sensitive and resistant cell lines is quite unlike the differential gene expression seen in the cell lines obtained using tyrosine kinase inhibitors. This agrees well with our previous study documenting that the transcriptional responses to EGFR antibody inhibitors are different from those to kinase inhibitors [9]. In sensitive cell lines, ontological categories related to immunity, cell proliferation, cell migration and response to external stimulus are overexpressed (Table 6a). In contrast, in the resistant cell lines epithelium development- and differentiation-associated gene ontologies are frequently and significantly overexpressed. Intriguing, the ontological category for wound response was overexpressed in resistant cells as well, although it had relatively less significant p-value.

**Table 6:** **Comparison of ontological categories differentially expressed in Cetuximab-sensitive** ***vs*** **. resistant cell lines**

	Cetuximab: Overexpressed in sensitive cells			Overexpressed in resistant cells	
	Term	p_value		Term	p_value
**Table 6a**					
**IM**	response to wounding	1.3E-10	**DF**	ectoderm development	9.2E-10
**CC**	regulation of cell proliferation	1.6E-09	**DF**	epidermis development	1.4E-09
**CC**	positive regulation of cell proliferation	3.5E-07	**DF**	epithelium development	1.0E-08
**M**	chemotaxis	8.9E-07	**DF**	epithelial cell differentiation	1.5E-07
**M**	taxis	8.9E-07	**DF**	keratinocyte differentiation	6.2E-05
**M**	cell migration	1.7E-06	**DF**	cornified envelope	6.5E-05
**M**	localization of cell	2.6E-06	**EC**	extracellular matrix	7.8E-05
**M**	cell motility	2.6E-06	**DF**	epidermal cell differentiation	1.2E-04
**M**	Cell communication	3.5E-06	**DF**	peptide cross-linking	1.7E-04
**R**	regulation of response to external stimulus	4.0E-06	**IM**	wound healing	1.7E-04
**Table 6b**					
**IM**	inflammatory response	6.5E-06	**CC**	regulation of cell proliferation	4.3E-04
**IM**	wound healing	9.1E-06	**CY**	Cell structure and motility	3.3E-04
**IM**	immune response	9.8E-06	**CY**	12q12-q13	6.9E-04
**IM**	Immunity and defense	6.7E-05	**T**	transcription repressor activity	8.4E-04
**IM**	regulation of inflammatory response	3.5E-04			
**S**	Signal transduction	1.5E-05			
**S**	Jak-STAT signaling pathway	5.1E-04			
**S**	JAK-STAT cascade	6.7E-04			
**V**	regulation of vascular endothelial growth factor production	3.2E-05			
**V**	Angiogenesis	3.8E-05			
**V**	blood coagulation	5.8E-04			

a) Top ten categories with best p-values. b) Selected categories with p-value better than 10^-4^ (see Additional file 7).

In Table 6b, the overregulated processes in Cetuximab-sensitive cell line comprise immunity responses, signal transduction involving JAK-STAT pathway and angiogenesis. In contrast, in the resistant cell line genes for cell cycle, cell structure and reduction of transcription are increased.

### Clustering of ontological categories in sensitive *vs.* resistant cell lines

The charts comparing overexpressed ontological categories, although very informative, contain many redundant and overlapping ontological categories (Tables 2,3,4,5 and 6). To get around this problem we have clustered the ontological categories using DAVID software. Cluster outputs for Erlotinib-, irreversible inhibitor and Cetuximab-sensitive versus resistant cell lines are presented in Additional file 8.

In Erlotinib-sensitive cell lines, transcription, translation and protein transport processes, marked as ‘T’ in Additional file 8a, are statistically more significant. In contrast, we found mitochondria-related genes, designated as ‘E’ for ‘energy’, the most frequent ontological category in resistant cell lines. Ontologies related to apoptosis, ‘A’, are the second most upregulated category in sensitive cells. In resistant cell lines genes related to cell cycle, ‘CC’, and cytoskeleton, ‘CY’, are the most expressed categories after energy production. In sensitive cell lines apparently energy is spent for synthesizing proteins and regulating cell death processes, whereas in resistant cells energy is mainly produced and utilized in cell growth maintenance. Thus, if this hypothesis is confirmed by the laboratory approaches, energy starvation strategies could be used to treat cancer cells.

Unexpectedly, categories ‘M’, describing motion of cells, and ‘V’, denoting the vascularization and angiogenesis, were found in sensitive cell lines, whereas adhesion-related genes, ‘AD’, were found relatively overexpressed in resistant cell lines. We have also found that certain categories related to energy metabolism; marked as ‘M-c’ for metabolism of carbohydrates and ‘E’ for energy-related processes are also up regulated in sensitive cells (Additional file 8a).

Although extracellular region ‘EC’ is the top regulated category in irreversible inhibitor-resistant cell lines, important EGFR-activated cellular processes, including immunity, ‘IM’, angiogenesis, ‘V’, and cytoskeleton, ‘CY’, were upregulated (Additional file 8b). In sensitive cell lines membrane systems, ‘MM’, response to hormones, ‘R’, and cell cycle, ‘CC’, were upregulated.

In Cetuximab antibody-resistant cell lines, many ontological categories are suppressed. For example, ontological category ‘IM’, immunity, is statistically the most prominent, biosynthesis of inflammatory cytokine and responses to these cytokines are specifically downregulated. We have observed that gene ontologies related to migration of the cells, marked ‘M’, are prominent in Additional file 8c. Cell adhesion categories, designated as ‘AD’, are also present there but the enrichment score and p-values are comparatively less. Blood vessels development processes, ‘V’, are overexpressed in Cetuximab-sensitive cell lines.

We have also found increased expression of signaling, ‘S’, apoptosis inhibition, ‘A’, and homeostasis-related genes, ‘H’, in sensitive cell lines, but not in resistant cell lines. Some of the overregulated clusters were also related to cell cycle, ‘CC’, transcription, ‘T’, and responses to external stimuli, ‘R’. Ectoderm development category was overexpressed with higher p-value in antibody-resistant cell lines, reflecting epidermal cell origin. Other ontological categories were extracellular matrix ‘EC’ and adhesion (Additional file 8c).

As observed above, (Tables 3,4,5 and 6), we have seen that responses of antibody-resistant cell line are reverse of those observed in tyrosine kinase inhibitors-resistant cell lines (comparing Additional files 8a and b with Table 6c). All ontological categories observed in Cetuximab-sensitive cell lines seem responsible for tumor cell survival and progression and were previously seen overexpressed in tyrosine kinase inhibitor-resistant cell lines.

## Discussion

Global metaanalysis of EGFR inhibitor-resistant *vs.* sensitive cell lines presented here identifies the transcriptional and metabolic differences that allow tumor cells to evade EGFR-targeted therapies. Specifically, we find that the most acute problem created by EGFR inhibition is to provide a sufficient energy supply to the proliferating and metabolizing transformed cells. This metabolic predicament for tumors was one of the first ones identified, already by Warburg in 1927 [28]. The energy deficit caused by inhibition of EGFR is overcome by several mechanisms, e.g., by major enhancement of the mitochondrial oxidative phosphorylation in Erlotinib-inhibited cells, or by promoting vascularization and angiogenesis in Gefitinib-inhibited tumors.

In aerobic glycolysis, tumor cells produce lactate by glycolysis in the presence of oxygen and decrease the oxidative phosphorylation due to presence of an isoform of pyruvate kinase. If pyruvate kinase is in its native form, then increased oxygen uptake and oxidative phosphorylation occur and lactate formation is decreased [29]. In metaanalysis of Erlotinib-resistant cells we found that mitochondrial processes involving oxidative phosphorylation were upregulated. Moreover, the resistant cells recruit supplementary energy sources, inducing enzymes that catabolize carbohydrates in addition to those catabolizing lipids.

Additionally, our study proposes that EGFR tyrosine kinase inhibitors-resistant cancer cell lines express specific inflammatory cytokines and angiogenesis signals to promote vascularization and perhaps autocrine immunity stimulation as important strategy to combat cancer treatment [13,30]. In-depth perusal of the processes enhanced in the resistant cell lines revealed involvement of interleukins and chemokines with C-X-C motif. These observations are in line with previously known facts that cancer cells use immune cells and cytokines in various ways to maintain their own proliferation [25]. This mode of overcoming EGFR inhibition is particularly prominent in cells selected for Gefitinib resistance.

Perhaps expectedly, while the sensitive cell lines depend on the ErbB-dependent signaling, resistant ones circumvent this pathway and, at least in some cases, rely on the Ras pathway. Parenthetically, the mutations that confer EGFR inhibitor resistance generally occur in EGFR, seldom in Ras. In targeted therapy, as is case of EGFR inhibition, small molecules targeting tyrosine kinase domain of EGFR or monoclonal antibodies specifically blocking the ligand binding site are used. In some tumors, ErbB-independent signaling is sustained by activation of downstream molecules, for example Ras, *PKI3CA* and *BRAF* [15,21,31-34]. Correspondingly, we find that in EGFR inhibitor-resistant cell lines, Ras-dependent oncogenic pathways are preferentially expressed as a strategy to overcome the EGFR inhibition (Table 2b). Detailed analysis of the ErbB and Ras pathway genes enhanced in the sensitive and resistant cell lines respectively, revealed that genes involved in these ontological categories are quite dissimilar (Table 2d).

Interestingly, we found that irreversible inhibitors of the EGFR signaling pathway increased in resistant cell lines the expression of multiple EGFR ligands (Table 5b,c). This seems to be an important alternative to other modes of overcoming EGFR inhibition [11,35].

Hypoxia stimulates various processes in cancer cells including vascularization, growth factor signaling, genetic instability, cell survival, metastasis, cell death and antitumor drug resistance, in general favoring tumor survival and propagation [36]. The cells in surrounding areas of low oxygen are considered resistant to antitumor drugs mainly due to non-availability of drug, change in responses to drug, induction of drug-resistant genes, increase in mutations, angiogenesis and metastasis genes enhancement [26]. Metaanalysis comparing data from Gefitinib-sensitive versus resistant cell lines revealed that gene ontologies responsive to lower oxygen levels are specifically upregulated in resistant cell lines thus favoring survival. This finding further supports our observation that use of different inhibitors results in different, specific strategies to resist the antitumor targeted therapy.

An important caveat in this analysis stems from the fact that few of the studies, namely GSE34228 and GSE38310 for Gefitinib and Erlotinib, respectively, dominate the metaanalysis by overwhelming numbers of microarrays. Therefore, we cannot claim at this point that it is the resistance to Gefitinib that specifically produces the described changes in all cell lines selected for resistance to Gefitinib, and *mutatis mutandis*, the same for Erlotinib. However, we note that both GSE34228 and GSE38310 compared Non-Small Cell Lung Cancer lines, so the characteristic differences between Gefitinib- *vs*. Erlotinib-selected resistance cannot be ascribed to different cell types. Additional data are needed to corroborate or refute inhibitor-specific characteristics of resistant cells. That said, our previous metaanalysis demonstrated that different inhibitors, although working by ostensibly same mechanisms, cause identifiably different transcriptional responses, which allows for inhibitor-specific developments of resistance as well.

## Conclusions

This metaanalysis of transcriptional and metabolic differences between EGFR inhibitor-resistant *vs.* sensitive cell lines identifies changes that allow tumor cells to evade EGFR-inhibition. The use of different inhibitors results in different, specific strategies to resist the antitumor therapy. Common pathways are upregulated in cell lines resistant to inhibitors targeting the kinase domain of EGFR, however, there are certain processes uniquely expressed against some of the inhibitors but, apparently, not others. The development of resistance to antibody inhibitors can vary significantly.

We found that the most acute problem created by EGFR inhibition is to provide a sufficient energy supply to the cells. The energy deficit can be overcome by several routes, e.g., by boosting mitochondrial oxidative phosphorylation, or by promoting vascularization and angiogenesis.

Further study of sensitive and resistant cancer cell lines responses to additional EGFR inhibitors will improve our understanding of drug resistance development and thus lead to improved anticancer treatment strategies. For example, use of mitochondrial blockers with Erlotinib, immunity blockers with Gefitinib, or combining tyrosine kinase inhibitors with antibody inhibitors, may avoid development of resistance to EGFR inhibitors.

## Additional files

Additional file 1: Figure S1. Flow chart of metaanalysis procedure.
Additional file 2: Figure S2. Differentially expressed genes in sensitive cell lines *vs.* resistant cell lines. Top panels genes overexpressed in resistant cell lines, bottom panels genes overexpressed in sensitive cell lines. a) All studies. b) Gefitinib. c) Erlotinib. d) Irreversible inhibitors. e) Cetuximab.
Additional file 3:
**Ontological categories characteristically expressed in EGFR inhibitors-sensitive** ***vs.*** **resistant cell lines.**

Additional file 4:
**Ontological categories characteristically expressed in Erlotinib-sensitive** ***vs.*** **resistant cell lines.**

Additional file 5:
**Ontological categories characteristically expressed in Gefitinib-sensitive** ***vs.*** **resistant cell lines.**

Additional file 6:
**Ontological categories characteristically expressed in irreversible inhibitors-sensitive** ***vs.*** **resistant cell lines.**

Additional file 7:
**Ontological categories characteristically expressed in Cetuximab-sensitive** ***vs.*** **resistant cell lines.**

Additional file 8: **a. Cluster analysis for Erlotinib Sensitive*****vs.*****regulated analysis using DAVID software.** For complete lists see Additional file 4. Additional files 8b and 8c Clusters of differentially expressed gene ontologies in irreversible inhibitor- and Cetuximab-sensitive *vs*. resistant cell lines, respectively. For complete lists see Additional files 6 and 7.

## References

[1] Vogelstein B, Kinzler KW (2004). Cancer genes and the pathways they control. Nat Med.

[2] Peto J, Houlston RS (2001). Genetics and the common cancers. Eur J Cancer.

[3] Vanneman M, Dranoff G (2012). Combining immunotherapy and targeted therapies in cancer treatment. Nat Rev Cancer.

[4] Hynes NE, Lane HA (2005). ERBB receptors and cancer: the complexity of targeted inhibitors. Nat Rev Cancer.

[5] Schlessinger J (2004). Common and distinct elements in cellular signaling via EGF and FGF receptors. Science.

[6] Blumenberg M (2013). Profiling and metaanalysis of epidermal keratinocytes responses to epidermal growth factor. BMC Genomics..

[7] Wheeler DL, Dunn EF, Harari PM (2010). Understanding resistance to EGFR inhibitors-impact on future treatment strategies. Nat Rev Clin Oncol.

[8] Li SN, Li HQ (2014). Epidermal growth factor receptor inhibitors: a patent review (2010 - present). Expert Opin Ther Pat.

[9] Blumenberg M (2014). Differential Transcriptional Effects of EGFR Inhibitors. PLoS One.

[10] Nakata A, Gotoh N (2012). Recent understanding of the molecular mechanisms for the efficacy and resistance of EGF receptor-specific tyrosine kinase inhibitors in non-small cell lung cancer. Expert Opin Ther Targets.

[11] Hatakeyama H, Cheng H, Wirth P, Counsell A, Marcrom SR, Wood CB (2010). Regulation of heparin-binding EGF-like growth factor by miR-212 and acquired cetuximab-resistance in head and neck squamous cell carcinoma. PLoS One.

[12] Cortot AB, Repellin CE, Shimamura T, Capelletti M, Zejnullahu K, Ercan D (2013). Resistance to irreversible EGF receptor tyrosine kinase inhibitors through a multistep mechanism involving the IGF1R pathway. Cancer Res.

[13] Zhang Z, Lee JC, Lin L, Olivas V, Au V, LaFramboise T (2012). Activation of the AXL kinase causes resistance to EGFR-targeted therapy in lung cancer. Nat Genet.

[14] Giles KM, Kalinowski FC, Candy PA, Epis MR, Zhang PM, Redfern AD (2013). Axl mediates acquired resistance of head and neck cancer cells to the epidermal growth factor receptor inhibitor erlotinib. Mol Cancer Ther.

[15] Chong CR, Janne PA (2013). The quest to overcome resistance to EGFR-targeted therapies in cancer. Nat Med.

[16] Gautier L, Cope L, Bolstad BM (2004). Irizarry RA: affy–analysis of Affymetrix GeneChip data at the probe level. Bioinformatics.

[17] Mimoso C, Lee DD, Zavadil J, Tomic-Canic M, Blumenberg M (2014). Analysis and meta-analysis of transcriptional profiling in human epidermis. Methods Mol Biol.

[18] Hong F, Breitling R, McEntee CW, Wittner BS, Nemhauser JL, Chory J (2006). RankProd: a bioconductor package for detecting differentially expressed genes in meta-analysis. Bioinformatics.

[19] Dennis G, Sherman BT, Hosack DA, Yang J, Gao W, Lane HC, Lempicki RA (2003). DAVID: Database for Annotation, Visualization, and Integrated Discovery. Genome Biol.

[20] Ohashi K, Sequist LV, Arcila ME, Moran T, Chmielecki J, Lin YL (2012). Lung cancers with acquired resistance to EGFR inhibitors occasionally harbor BRAF gene mutations but lack mutations in KRAS, NRAS, or MEK1. Proc Natl Acad Sci U S A.

[21] Fulda S, Gorman AM, Hori O, Samali A (2010). Cellular stress responses: cell survival and cell death. Int J Cell Biol.

[22] Ulrich CM, Bigler J, Potter JD (2006). Non-steroidal anti-inflammatory drugs for cancer prevention: promise, perils and pharmacogenetics. Nat Rev Cancer.

[23] Yap TA, Sandhu SK, Carden CP, de Bono JS (2011). Poly (ADP-ribose) polymerase (PARP) inhibitors: Exploiting a synthetic lethal strategy in the clinic. CA Cancer J Clin.

[24] Mimeault M, Batra SK (2007). Functions of tumorigenic and migrating cancer progenitor cells in cancer progression and metastasis and their therapeutic implications. Cancer Metastasis Rev.

[25] Dranoff G (2004). Cytokines in cancer pathogenesis and cancer therapy. Nat Rev Cancer.

[26] Brown JM, Wilson WR (2004). Exploiting tumour hypoxia in cancer treatment. Nat Rev Cancer.

[27] Boeckx C, Op de Beeck K, Wouters A, Deschoolmeester V, Limame R, Zwaenepoel K (2014). Overcoming cetuximab resistance in HNSCC: The role of AURKB and DUSP proteins. Cancer Lett.

[28] Warburg O, Wind F, Negelein E (1927). The metabolism of tumors in the body. J Gen Physiol.

[29] Christofk HR, Vander Heiden MG, Harris MH, Ramanathan A, Gerszten RE, Wei R (2008). The M2 splice isoform of pyruvate kinase is important for cancer metabolism and tumour growth. Nature.

[30] Luo J, Solimini NL, Elledge SJ (2009). Principles of cancer therapy: oncogene and non-oncogene addiction. Cell.

[31] Diaz LA, Williams RT, Wu J, Kinde I, Hecht JR, Berlin J (2012). The molecular evolution of acquired resistance to targeted EGFR blockade in colorectal cancers. Nature.

[32] Misale S, Yaeger R, Hobor S, Scala E, Janakiraman M, Liska D (2012). Emergence of KRAS mutations and acquired resistance to anti-EGFR therapy in colorectal cancer. Nature.

[33] Sartore-Bianchi A, Martini M, Molinari F, Veronese S, Nichelatti M, Artale S (2009). PIK3CA mutations in colorectal cancer are associated with clinical resistance to EGFR-targeted monoclonal antibodies. Cancer Res.

[34] Lievre A, Bachet JB, Le Corre D, Boige V, Landi B, Emile JF (2006). KRAS mutation status is predictive of response to cetuximab therapy in colorectal cancer. Cancer Res.

[35] Rajput A, Koterba AP, Kreisberg JI, Foster JM, Willson JK, Brattain MG (2007). A novel mechanism of resistance to epidermal growth factor receptor antagonism in vivo. Cancer Res.

[36] Harris AL (2002). Hypoxia–a key regulatory factor in tumour growth. Nat Rev Cancer.

