# Supplementary figures and images for: Transcriptional changes associated with resistance to inhibitors of epidermal growth factor receptor revealed using metaanalysis
Source: BMC Cancer. 2015 May 7;15:369. doi: 10.1186/s12885-015-1337-3 (PMC4430867; doi:10.1186/s12885-015-1337-3)

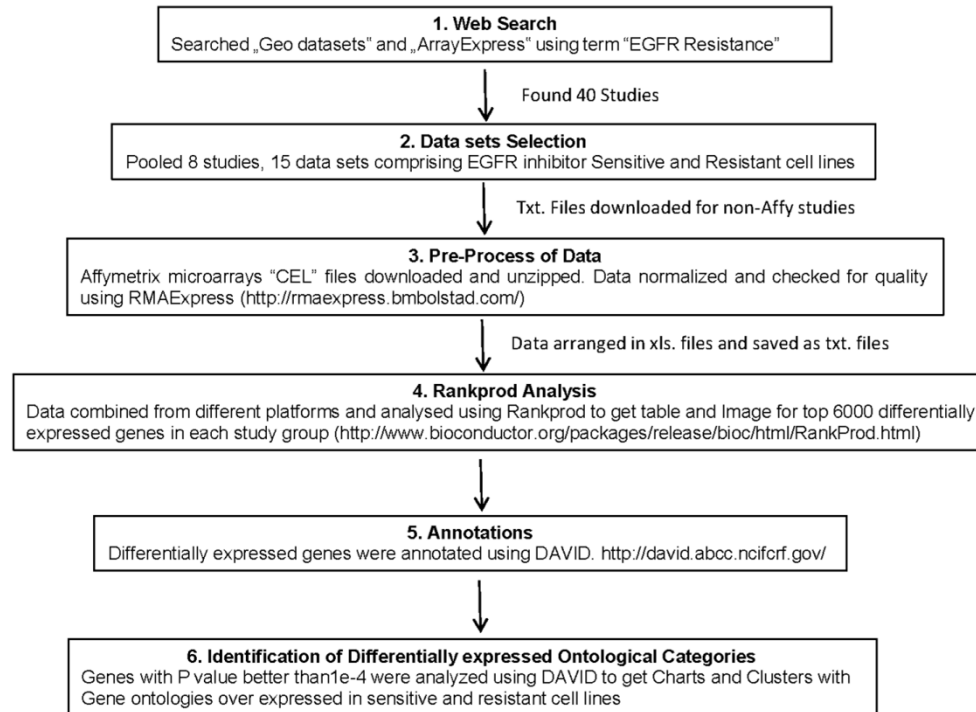

Figure 1. Flow chart of metaanalysis procedure.

Supplement: Additional file 1: Figure S1. — Flow chart of metaanalysis procedure. [file 12885_2015_1337_MOESM1_ESM.pdf]
