# Supplementary material for: Transcriptional changes associated with resistance to inhibitors of epidermal growth factor receptor revealed using metaanalysis
Source: BMC Cancer. 2015 May 7;15:369. doi: 10.1186/s12885-015-1337-3 (PMC4430867; doi:10.1186/s12885-015-1337-3)
Supplement: Additional file 2: Figure S2. — Differentially expressed genes in sensitive cell lines vs. resistant cell lines. Top panels genes overexpressed in resistant cell lines, bottom panels genes overexpressed in sensitive cell lines. a) All studies. b) Gefitinib. c) Erlotinib. d) Irreversible inhibitors. e) Cetuximab. [file 12885_2015_1337_MOESM2_ESM.pdf]

Figure 2. Differentially expressed genes in sensitive cell lines *vs.* resistant cell lines. Top panels genes overexpressed in resistant cell lines, bottom panels genes overexpressed in sensitive cell lines. a) All studies. b) Gefitinib. c) Erlotinib. d) Irreversible inhibitors. e) Cetuximab.

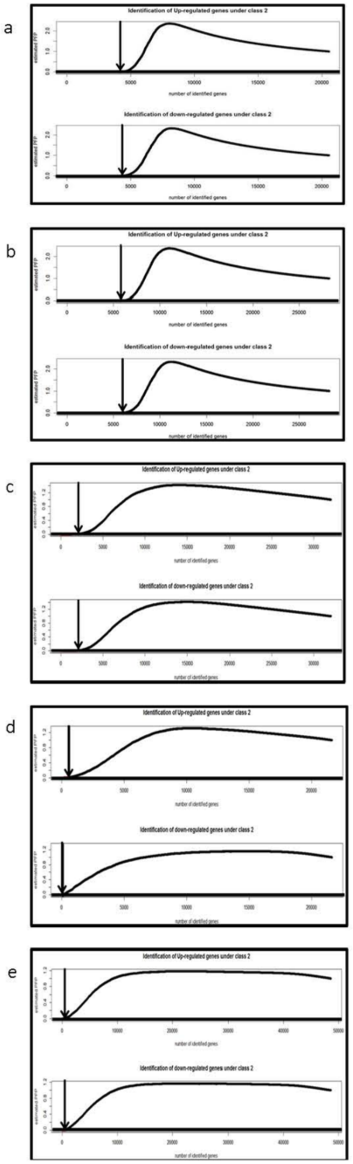

Figure 2: Differentially expressed genes in Sensitive cell lines vs Resistant cell lines a. All Studies b. Gefitinib c. Erlotinib d. Irreversible e. Cetuximab
