# Supplementary material for: Transcriptional changes associated with resistance to inhibitors of epidermal growth factor receptor revealed using metaanalysis
Source: BMC Cancer. 2015 May 7;15:369. doi: 10.1186/s12885-015-1337-3 (PMC4430867; doi:10.1186/s12885-015-1337-3)
Supplement: Additional file 3: — Ontological categories characteristically expressed in EGFR inhibitors-sensitive vs. resistant cell lines. [file 12885_2015_1337_MOESM3_ESM.zip › 12885_2015_1337_add3.pdf]

All studies: Overexpressed in sensitive cells	
**Supplement 3**	
**Term**	**p_Value**
Pathways in cancer	4.9E-15
plasma membrane part	7.7E-12
regulation of cell proliferation	1.3E-10
endomembrane system	1.4E-10
cell fraction	2.1E-09
regulation of cell death	2.2E-09
regulation of programmed cell death	2.6E-09
positive regulation of macromolecule metabolic	2.6E-09
cell proliferation	5.7E-09
protein complex biogenesis	7.0E-09
protein complex assembly	7.0E-09
organelle lumen	1.0E-08
regulation of apoptosis	1.3E-08
membrane-enclosed lumen	1.5E-08
nuclear lumen	2.0E-08
Pancreatic cancer	2.8E-08
positive regulation of molecular function	3.4E-08
macromolecular complex subunit organization	3.5E-08
cell migration	5.0E-08
regulation of transcription from RNA polymeras	5.3E-08
macromolecular complex assembly	5.6E-08
intracellular organelle lumen	5.6E-08
cytosol	5.7E-08
positive regulation of cell proliferation	6.5E-08
cell motion	7.7E-08
vasculature development	8.8E-08
positive regulation of transcription from RNA pc	1.1E-07
intracellular signaling cascade	1.1E-07
Bladder cancer	1.2E-07
negative regulation of cell death	1.4E-07
membrane fraction	1.7E-07
integral to plasma membrane	2.0E-07
cell adhesion	2.3E-07
negative regulation of programmed cell death	2.3E-07
biological adhesion	2.5E-07
blood vessel development	2.8E-07
positive regulation of programmed cell death	2.9E-07
positive regulation of nitrogen compound metak	2.9E-07
positive regulation of developmental process	3.1E-07
insoluble fraction	3.1E-07
positive regulation of macromolecule biosynthe	3.3E-07
positive regulation of biosynthetic process	3.4E-07
intrinsic to plasma membrane	3.6E-07
positive regulation of cell death	3.8E-07
identical protein binding	3.9E-07
in utero embryonic development	4.0E-07
positive regulation of cellular biosynthetic proce	5.0E-07
enzyme binding	5.8E-07
positive regulation of apoptosis	6.1E-07
positive regulation of nucleobase, nucleoside, n	6.2E-07

transcription activator activity	6.4E-07
negative regulation of apoptosis	7.1E-07
regulation of phosphorus metabolic process	8.7E-07
regulation of phosphate metabolic process	8.7E-07
positive regulation of transcription, DNA-depend	9.7E-07
phosphorus metabolic process	1.0E-06
phosphate metabolic process	1.0E-06
regulation of cellular protein metabolic process	1.1E-06
cell motility	1.2E-06
localization of cell	1.2E-06
positive regulation of gene expression	1.2E-06
regulation of phosphorylation	1.2E-06
positive regulation of transcription	1.3E-06
positive regulation of RNA metabolic process	1.6E-06
kinase binding	1.6E-06
response to organic substance	1.9E-06
positive regulation of catalytic activity	2.4E-06
homeostatic process	2.4E-06
regulation of protein amino acid phosphorylation	2.6E-06
negative regulation of macromolecule metaboli	2.7E-06
cell projection	3.2E-06
intracellular non-membrane-bounded organelle	3.3E-06
non-membrane-bounded organelle	3.3E-06
anti-apoptosis	3.3E-06
nucleolus	3.5E-06
nucleotide binding	3.5E-06
Colorectal cancer	3.8E-06
regulation of protein modification process	4.8E-06
positive regulation of cell differentiation	5.3E-06
regulation of lipid metabolic process	6.8E-06
chemical homeostasis	7.0E-06
phosphorylation	7.0E-06
3p21.3	7.3E-06
protein dimerization activity	8.0E-06
regulation of transferase activity	8.4E-06
purine nucleotide binding	8.6E-06
Intracellular signaling cascade	8.9E-06
regulation of protein kinase cascade	9.4E-06
regulation of locomotion	1.0E-05
placenta development	1.1E-05
cellular cation homeostasis	1.1E-05
chordate embryonic development	1.1E-05
protein amino acid phosphorylation	1.2E-05
response to inorganic substance	1.2E-05
perinuclear region of cytoplasm	1.2E-05
cell death	1.3E-05
purine nucleoside binding	1.4E-05
cell cycle	1.4E-05
Leukocyte transendothelial migration	1.5E-05
response to abiotic stimulus	1.5E-05
protein kinase binding	1.7E-05
embryonic development ending in birth or egg h	1.7E-05
cell activation	1.8E-05
Protein phosphorylation	1.8E-05
membrane-bounded vesicle	1.8E-05
adenyl nucleotide binding	1.9E-05

regulation of kinase activity	2.0E-05
death	2.0E-05
nucleoside binding	2.0E-05
vesicle	2.1E-05
cell-cell signaling	2.1E-05
forebrain development	2.2E-05
cell leading edge	2.3E-05
cell cycle process	2.5E-05
regulation of cell motion	2.5E-05
regulation of cell migration	2.7E-05
cytoplasmic membrane-bounded vesicle	3.2E-05
protein kinase cascade	3.3E-05
Oncogenesis	3.4E-05
behavior	3.6E-05
T cell receptor signaling pathway	3.7E-05
purine ribonucleotide binding	3.7E-05
ribonucleotide binding	3.7E-05
intracellular transport	3.7E-05
positive regulation of protein amino acid phospl	3.9E-05
negative regulation of macromolecule biosynthe	3.9E-05
response to ethanol	4.2E-05
leukocyte activation	4.2E-05
Focal adhesion	4.3E-05
response to wounding	4.4E-05
5q31-q32	4.5E-05
negative regulation of cellular protein metabolic	4.5E-05
enzyme linked receptor protein signaling pathwa	4.6E-05
extracellular region part	4.7E-05
11q13	5.1E-05
negative regulation of biosynthetic process	5.2E-05
blastocyst formation	5.3E-05
ATP binding	5.5E-05
establishment of protein localization	5.5E-05
cellular di-, tri-valent inorganic cation homeosta	5.5E-05
positive regulation of locomotion	5.7E-05
11p15.5	5.7E-05
regulation of binding	5.9E-05
positive regulation of response to stimulus	6.0E-05
negative regulation of cellular biosynthetic proc	6.1E-05
membrane raft	6.1E-05
negative regulation of phosphate metabolic pro	6.2E-05
negative regulation of phosphorus metabolic pr	6.2E-05
Neurotrophin signaling pathway	6.4E-05
regulation of protein kinase activity	6.6E-05
lymphocyte activation	6.8E-05
negative regulation of phosphorylation	7.0E-05
response to drug	7.1E-05
phosphoinositide metabolic process	7.3E-05
nucleoplasm	7.3E-05
negative regulation of protein metabolic process	7.4E-05
protein localization	7.4E-05
cytoplasmic vesicle	7.7E-05
positive regulation of cell communication	7.9E-05
negative regulation of molecular function	8.5E-05
leukocyte differentiation	8.6E-05
cation homeostasis	8.7E-05

positive regulation of protein metabolic process	8.9E-05
nuclear envelope	8.9E-05
negative regulation of cell proliferation	9.0E-05
positive regulation of signal transduction	9.0E-05
positive regulation of phosphorus metabolic pro	9.1E-05
positive regulation of phosphate metabolic proc	9.1E-05
cellular component morphogenesis	9.2E-05
organelle membrane	9.4E-05
adenyl ribonucleotide binding	9.5E-05
protein transport	9.5E-05
positive regulation of cell migration	1.0E-04
Chronic myeloid leukemia	1.0E-04
positive regulation of lipid metabolic process	1.0E-04
pallium development	1.0E-04
cerebral cortex development	1.0E-04
10q24	1.1E-04
positive regulation of phosphorylation	1.1E-04
lipid biosynthetic process	1.1E-04
blood vessel morphogenesis	1.2E-04
regulation of hydrolase activity	1.2E-04
protein homodimerization activity	1.3E-04
response to oxygen levels	1.3E-04
response to metal ion	1.3E-04
cellular protein complex assembly	1.4E-04
positive regulation of protein modification proce	1.4E-04
positive regulation of immune system process	1.5E-04
regulation of DNA metabolic process	1.5E-04
organophosphate metabolic process	1.5E-04
14q12-q13	1.5E-04
transcription factor binding	1.5E-04
Kinase	1.5E-04
response to steroid hormone stimulus	1.6E-04
Protein kinase	1.6E-04
telencephalon development	1.7E-04
di-, tri-valent inorganic cation homeostasis	1.7E-04
leukocyte proliferation	1.7E-04
mononuclear cell proliferation	1.7E-04
22q13.1	1.8E-04
response to nutrient levels	1.8E-04
collagen metabolic process	1.9E-04
activation of pro-apoptotic gene products	1.9E-04
programmed cell death	1.9E-04
Golgi apparatus	2.0E-04
Protein modification	2.1E-04
Melanoma	2.2E-04
phospholipid metabolic process	2.3E-04
response to extracellular stimulus	2.3E-04
tube development	2.3E-04
positive regulation of cellular protein metabolic	2.4E-04
defense response	2.6E-04
soluble fraction	2.7E-04
negative regulation of protein amino acid phosp	2.7E-04
apoptosis	2.8E-04
lymphocyte proliferation	2.8E-04
forebrain cell migration	2.9E-04
Prostate cancer	3.0E-04

cytoplasmic vesicle part	3.0E-04
lamellipodium	3.1E-04
3q21	3.1E-04
extracellular space	3.2E-04
positive regulation of protein kinase cascade	3.2E-04
positive regulation of multicellular organismal p	3.2E-04
response to hypoxia	3.2E-04
positive regulation of cell motion	3.2E-04
external side of plasma membrane	3.2E-04
endoplasmic reticulum	3.3E-04
fat cell differentiation	3.4E-04
regulation of smooth muscle cell proliferation	3.4E-04
hemopoiesis	3.5E-04
plasma membrane	3.7E-04
regulation of peptidyl-tyrosine phosphorylation	3.8E-04
tube morphogenesis	4.0E-04
blastocyst development	4.0E-04
regulation of cell growth	4.1E-04
Angiogenesis	4.1E-04
cellular chemical homeostasis	4.1E-04
negative regulation of catalytic activity	4.2E-04
cellular lipid catabolic process	4.2E-04
ErbB signaling pathway	4.2E-04
Adherens junction	4.5E-04
Small cell lung cancer	4.7E-04
vesicle-mediated transport	4.8E-04
respiratory system development	4.9E-04
vesicle membrane	5.0E-04
RNA polymerase II transcription factor activity	5.0E-04
cellular metal ion homeostasis	5.4E-04
multicellular organismal metabolic process	5.4E-04
glycerolipid metabolic process	5.4E-04
response to acid	5.4E-04
positive regulation of hydrolase activity	5.4E-04
cellular ion homeostasis	5.6E-04
cell morphogenesis	5.7E-04
response to corticosteroid stimulus	5.7E-04
mitotic cell cycle	5.9E-04
Acute myeloid leukemia	6.0E-04
Immunity and defense	6.1E-04
immune system development	6.2E-04
regulation of cell size	6.4E-04
regulation of response to external stimulus	6.5E-04
positive regulation of binding	6.8E-04
T cell activation	6.8E-04
Endometrial cancer	7.0E-04
receptor metabolic process	7.0E-04
multicellular organismal macromolecule metabo	7.0E-04
oligosaccharide metabolic process	7.0E-04
membrane organization	7.0E-04
cytosolic calcium ion homeostasis	7.0E-04
glycerophospholipid metabolic process	7.0E-04
regulation of cytokine production	7.2E-04
Axon guidance	7.4E-04
Apoptosis	7.5E-04
hemopoietic or lymphoid organ development	7.6E-04

Wnt signaling pathway	7.7E-04
small GTPase mediated signal transduction	7.9E-04
regulation of isotype switching	8.1E-04
General vesicle transport	8.1E-04
locomotory behavior	8.2E-04
cellular homeostasis	8.2E-04
angiogenesis	8.3E-04
humoral immune response	8.4E-04
negative regulation of axonogenesis	8.6E-04
lipoprotein binding	8.6E-04
regulation of isotype switching to IgG isotypes	8.7E-04
cell surface	8.7E-04
positive regulation of gene-specific transcriptior	8.8E-04
protein complex binding	8.8E-04
adherens junction	9.0E-04
signal complex assembly	9.2E-04
trophectodermal cell differentiation	9.2E-04
telencephalon cell migration	9.2E-04
lipoprotein particle clearance	9.2E-04
protein kinase activity	9.4E-04
Other extracellular matrix	9.4E-04
endocytic vesicle	9.5E-04
inflammatory response	9.6E-04
cellular macromolecular complex subunit organ	9.7E-04
internal side of plasma membrane	9.7E-04
positive regulation of transferase activity	9.8E-04
negative regulation of transcription	1.0E-03

Overexpressed in resistant cells

Term	p_Value
positive regulation of biosynthetic process	3.6E-17
positive regulation of cellular biosynthetic proces	5.2E-17
positive regulation of macromolecule metabolic	2.3E-16
response to endogenous stimulus	8.9E-16
positive regulation of nitrogen compound metab	1.6E-15
positive regulation of macromolecule biosynthet	9.3E-15
regulation of cell proliferation	4.9E-14
response to hormone stimulus	6.9E-14
response to organic substance	9.6E-14
positive regulation of nucleobase, nucleoside, nu	1.2E-13
positive regulation of transcription	1.0E-12
positive regulation of gene expression	1.2E-12
positive regulation of transcription, DNA-depend	2.6E-12
positive regulation of RNA metabolic process	2.6E-12
regulation of cell motion	2.8E-12
response to steroid hormone stimulus	6.2E-12
regulation of cell migration	8.4E-12
regulation of locomotion	1.8E-11
cell fraction	4.1E-11
response to nutrient levels	7.9E-11
response to drug	1.1E-10
positive regulation of cell proliferation	1.1E-10
positive regulation of locomotion	1.3E-10
positive regulation of cell motion	1.3E-10
positive regulation of cell migration	1.4E-10
cytosol	1.7E-10
positive regulation of molecular function	2.6E-10
Pathways in cancer	2.8E-10
regulation of programmed cell death	3.0E-10
cell activation	3.1E-10
response to extracellular stimulus	3.1E-10
regulation of apoptosis	3.3E-10
transmembrane receptor protein tyrosine kinase	3.4E-10
negative regulation of apoptosis	4.1E-10
regulation of cell death	4.4E-10
hemopoietic or lymphoid organ development	6.4E-10
enzyme binding	9.1E-10
intracellular signaling cascade	9.6E-10
negative regulation of programmed cell death	1.1E-09
membrane-enclosed lumen	1.1E-09
negative regulation of cell death	1.3E-09
response to oxygen levels	1.8E-09
immune system development	1.8E-09
leukocyte activation	1.8E-09
organelle lumen	2.0E-09
hemopoiesis	2.6E-09
positive regulation of developmental process	2.7E-09
nuclear lumen	3.0E-09
response to nutrient	3.8E-09
positive regulation of transcription from RNA pol	4.6E-09

response to hypoxia	5.1E-09
regulation of transcription from RNA polymerase	8.4E-09
identical protein binding	9.8E-09
intracellular organelle lumen	1.0E-08
membrane fraction	1.0E-08
response to peptide hormone stimulus	1.1E-08
response to inorganic substance	1.1E-08
nucleoplasm	1.1E-08
negative regulation of macromolecule metabolic	1.2E-08
insoluble fraction	1.4E-08
cell motion	2.3E-08
response to wounding	2.4E-08
response to estrogen stimulus	2.6E-08
regulation of phosphorylation	3.7E-08
regulation of neuron apoptosis	4.5E-08
cytoskeleton organization	6.1E-08
circulatory system process	6.1E-08
blood circulation	6.1E-08
regulation of cellular protein metabolic process	6.4E-08
anti-apoptosis	8.1E-08
regulation of cytokine production	1.1E-07
regulation of response to external stimulus	1.1E-07
enzyme linked receptor protein signaling pathwa	1.2E-07
response to insulin stimulus	1.5E-07
regulation of cell activation	1.5E-07
cell-cell signaling	1.5E-07
regulation of phosphorus metabolic process	1.6E-07
regulation of phosphate metabolic process	1.6E-07
cell proliferation	2.0E-07
homeostatic process	2.0E-07
positive regulation of binding	2.3E-07
regulation of lipid metabolic process	2.6E-07
positive regulation of cellular component organiz	2.6E-07
extracellular region part	2.6E-07
vasculature development	3.0E-07
plasma membrane part	3.0E-07
regulation of protein modification process	3.1E-07
leukocyte differentiation	3.8E-07
blood vessel development	4.2E-07
positive regulation of DNA binding	4.5E-07
positive regulation of catalytic activity	5.4E-07
response to abiotic stimulus	5.5E-07
protein complex assembly	7.1E-07
protein complex biogenesis	7.1E-07
regulation of cell adhesion	7.1E-07
positive regulation of programmed cell death	7.5E-07
positive regulation of cell communication	7.7E-07
positive regulation of cell differentiation	8.5E-07
response to corticosteroid stimulus	8.9E-07
positive regulation of cell death	9.7E-07
aging	1.1E-06
response to vitamin	1.2E-06
response to molecule of bacterial origin	1.3E-06
regulation of protein amino acid phosphorylation	1.3E-06
Thyroid cancer	1.4E-06
positive regulation of transcription factor activity	1.4E-06

regulation of leukocyte activation	1.5E-06
response to metal ion	1.5E-06
positive regulation of apoptosis	1.5E-06
response to lipopolysaccharide	1.8E-06
promoter binding	1.9E-06
regulation of epithelial cell proliferation	2.3E-06
protein kinase cascade	2.4E-06
cellular response to insulin stimulus	2.6E-06
response to glucocorticoid stimulus	2.6E-06
chemical homeostasis	2.7E-06
cell migration	2.9E-06
cellular chemical homeostasis	3.0E-06
cell projection	3.1E-06
cytoskeletal protein binding	3.2E-06
Focal adhesion	3.2E-06
lymphocyte differentiation	3.3E-06
cell morphogenesis	3.5E-06
regulation of protein kinase cascade	3.6E-06
tissue remodeling	3.7E-06
regulation of blood pressure	3.8E-06
negative regulation of cell proliferation	3.9E-06
protein complex binding	4.0E-06
cell motility	4.1E-06
localization of cell	4.1E-06
cellular ion homeostasis	4.1E-06
regulation of lymphocyte activation	4.1E-06
Adherens junction	4.3E-06
positive regulation of immune system process	4.3E-06
monosaccharide metabolic process	4.3E-06
positive regulation of signal transduction	4.3E-06
regulation of angiogenesis	4.6E-06
lymphocyte activation	4.9E-06
blood vessel morphogenesis	5.1E-06
cellular component morphogenesis	5.2E-06
regulation of binding	5.4E-06
regulation of system process	5.5E-06
regulation of transferase activity	5.6E-06
extracellular space	5.6E-06
positive regulation of multicellular organismal pr	6.0E-06
positive regulation of response to stimulus	6.0E-06
negative regulation of cell differentiation	6.1E-06
response to estradiol stimulus	6.1E-06
integral to plasma membrane	6.4E-06
negative regulation of neuron apoptosis	6.6E-06
regulation of cytokine biosynthetic process	6.6E-06
regulation of kinase activity	7.2E-06
regulation of T cell activation	7.5E-06
ion homeostasis	8.1E-06
response to organic cyclic substance	8.2E-06
cell cycle	8.2E-06
actin cytoskeleton organization	8.6E-06
regulation of cellular component size	9.0E-06
response to reactive oxygen species	9.2E-06
cell-matrix adhesion	9.5E-06
positive regulation of kinase activity	1.0E-05
protein dimerization activity	1.2E-05

hexose metabolic process	1.2E-05
response to hydrogen peroxide	1.4E-05
regulation of protein kinase activity	1.4E-05
actin filament-based process	1.4E-05
cellular homeostasis	1.5E-05
vesicle-mediated transport	1.5E-05
intrinsic to plasma membrane	1.5E-05
negative regulation of transport	1.6E-05
response to organic nitrogen	1.6E-05
cell-substrate adhesion	1.6E-05
negative regulation of biosynthetic process	1.7E-05
Small cell lung cancer	1.7E-05
negative regulation of macromolecule biosynthe	1.8E-05
Cell cycle control	1.9E-05
di-, tri-valent inorganic cation homeostasis	1.9E-05
positive regulation of protein kinase activity	2.0E-05
positive regulation of transferase activity	2.2E-05
regulation of growth	2.5E-05
transcription activator activity	2.5E-05
positive regulation of cell activation	2.6E-05
inflammatory response	2.8E-05
kinase binding	2.8E-05
regulation of organelle organization	2.8E-05
regulation of gene-specific transcription	2.9E-05
cellular cation homeostasis	2.9E-05
neuron projection	3.0E-05
cellular response to stress	3.1E-05
response to cytokine stimulus	3.2E-05
regulation of cell size	3.4E-05
regulation of cell cycle	3.5E-05
8p11.2	3.5E-05
regeneration	3.7E-05
cellular di-, tri-valent inorganic cation homeostas	3.8E-05
positive regulation of gene-specific transcription	4.0E-05
non-membrane-bounded organelle	4.2E-05
intracellular non-membrane-bounded organelle	4.2E-05
myeloid leukocyte activation	4.4E-05
cell cycle process	4.4E-05
protein homodimerization activity	4.6E-05
negative regulation of cellular biosynthetic proce	4.6E-05
muscle cell differentiation	4.7E-05
regulation of DNA binding	4.7E-05
regulation of leukocyte proliferation	4.7E-05
regulation of mononuclear cell proliferation	4.7E-05
positive regulation of cellular protein metabolic p	5.1E-05
regulation of oxidoreductase activity	5.1E-05
liver development	5.2E-05
cellular response to hormone stimulus	5.2E-05
regulation of transcription factor activity	5.6E-05
membrane-bounded vesicle	5.7E-05
glucose metabolic process	5.8E-05
cytoplasmic membrane-bounded vesicle	5.9E-05
T cell activation	6.0E-05
nucleoplasm part	6.1E-05
positive regulation of protein metabolic process	6.5E-05
macromolecular complex subunit organization	6.7E-05

positive regulation of response to external stimu	6.8E-05
extracellular matrix organization	7.1E-05
positive regulation of transport	7.3E-05
regulation of cellular localization	7.4E-05
cation homeostasis	7.5E-05
respiratory system development	7.5E-05
negative regulation of gene expression	7.6E-05
Non-receptor tyrosine protein kinase	7.8E-05
positive regulation of lymphocyte activation	8.0E-05
Prostate cancer	8.0E-05
chromatin modification	8.3E-05
positive regulation of leukocyte migration	8.3E-05
positive regulation of protein kinase cascade	8.5E-05
11p15.5	8.6E-05
positive regulation of cytokine production	8.8E-05
regulation of immunoglobulin production	9.0E-05
axon	9.0E-05
RNA biosynthetic process	9.2E-05
regulation of striated muscle cell differentiation	9.2E-05
membrane raft	9.3E-05
positive regulation of cytokine biosynthetic proce	9.4E-05
transcription, DNA-dependent	9.5E-05
protein tyrosine kinase activity	9.5E-05
regulation of lymphocyte proliferation	9.6E-05
actin cytoskeleton	1.0E-04
Intracellular signaling cascade	1.0E-04
negative regulation of multicellular organismal p	1.0E-04
Viral myocarditis	1.0E-04
macromolecular complex assembly	1.0E-04
regulation of lipid biosynthetic process	1.1E-04
negative regulation of phosphorylation	1.1E-04
respiratory tube development	1.1E-04
positive regulation of leukocyte activation	1.1E-04
Colorectal cancer	1.2E-04
phosphorylation	1.2E-04
regulation of DNA metabolic process	1.2E-04
extrinsic to membrane	1.3E-04
neuron differentiation	1.3E-04
cytoplasmic vesicle	1.3E-04
lung development	1.3E-04
phosphate metabolic process	1.3E-04
phosphorus metabolic process	1.3E-04
Bladder cancer	1.3E-04
Pancreatic cancer	1.4E-04
locomotory behavior	1.4E-04
19q13.4	1.4E-04
regulation of smooth muscle cell proliferation	1.5E-04
negative regulation of transcription from RNA pc	1.5E-04
regulation of nitric oxide biosynthetic process	1.5E-04
cell projection morphogenesis	1.6E-04
wound healing	1.6E-04
tube development	1.6E-04
positive regulation of protein modification proces	1.6E-04
gland development	1.7E-04
negative regulation of cell adhesion	1.7E-04
immune response	1.8E-04

vesicle	1.8E-04
skeletal system development	1.9E-04
calcium ion homeostasis	1.9E-04
cell adhesion	1.9E-04
behavior	1.9E-04
regulation of cellular ketone metabolic process	2.0E-04
regulation of cell development	2.0E-04
negative regulation of nitrogen compound metal	2.0E-04
angiogenesis	2.0E-04
biological adhesion	2.0E-04
intracellular receptor-mediated signaling pathwa	2.1E-04
Ras protein signal transduction	2.1E-04
cell projection organization	2.1E-04
Endometrial cancer	2.2E-04
neuron projection development	2.2E-04
cell part morphogenesis	2.2E-04
regulation of peptidyl-tyrosine phosphorylation	2.2E-04
cytoskeleton	2.2E-04
protein kinase binding	2.2E-04
regulation of endocytosis	2.3E-04
regulation of establishment of protein localizatio	2.3E-04
negative regulation of transcription, DNA-depend	2.4E-04
response to temperature stimulus	2.4E-04
positive regulation of smooth muscle cell prolifer	2.4E-04
regulation of receptor biosynthetic process	2.5E-04
neuron development	2.5E-04
basolateral plasma membrane	2.5E-04
regulation of endothelial cell migration	2.6E-04
mitotic cell cycle	2.6E-04
Signaling molecule	2.7E-04
positive regulation of T cell activation	2.7E-04
negative regulation of RNA metabolic process	2.8E-04
death	2.9E-04
positive regulation of growth	2.9E-04
regulation of cellular component biogenesis	2.9E-04
negative regulation of phosphorus metabolic prc	3.1E-04
negative regulation of phosphate metabolic proc	3.1E-04
cellular calcium ion homeostasis	3.1E-04
p53 pathway feedback loops 2	3.1E-04
non-membrane spanning protein tyrosine kinase	3.2E-04
striated muscle cell differentiation	3.3E-04
cellular metal ion homeostasis	3.3E-04
intracellular transport	3.3E-04
neuron projection morphogenesis	3.5E-04
protein amino acid phosphorylation	3.5E-04
regulation of vesicle-mediated transport	3.6E-04
heterocycle biosynthetic process	3.6E-04
negative regulation of nucleobase, nucleoside, r	3.6E-04
4q21	3.7E-04
metal ion homeostasis	3.7E-04
negative regulation of response to stimulus	3.7E-04
transcription factor complex	3.7E-04
Cell cycle	3.7E-04
response to osmotic stress	3.8E-04
cytokine-mediated signaling pathway	3.8E-04
cell death	3.8E-04

response to oxidative stress	3.9E-04
Allograft rejection	4.0E-04
Oncogenesis	4.0E-04
regulation of skeletal muscle tissue developmen	4.0E-04
negative regulation of cell motion	4.0E-04
regulation of leukocyte migration	4.1E-04
regulation of cytoskeleton organization	4.1E-04
nucleoside binding	4.1E-04
cell morphogenesis involved in differentiation	4.2E-04
positive regulation of angiogenesis	4.2E-04
positive regulation of oxidoreductase activity	4.2E-04
organ regeneration	4.2E-04
regulation of DNA recombination	4.2E-04
positive regulation of mononuclear cell proliferat	4.2E-04
positive regulation of leukocyte proliferation	4.2E-04
integrin binding	4.2E-04
regulation of fatty acid metabolic process	4.2E-04
microsome	4.3E-04
Signaling in Immune system	4.3E-04
positive regulation of phosphorylation	4.4E-04
cofactor biosynthetic process	4.4E-04
defense response	4.5E-04
regulation of smooth muscle cell migration	4.5E-04
regulation of muscle cell differentiation	4.5E-04
regulation of immune effector process	4.5E-04
soluble fraction	4.8E-04
positive regulation of lipid metabolic process	4.9E-04
Angiogenesis	5.0E-04
purine nucleoside binding	5.0E-04
protein oligomerization	5.1E-04
cofactor metabolic process	5.2E-04
positive regulation of cell cycle	5.6E-04
negative regulation of response to external stimu	5.8E-04
regulation of striated muscle tissue developmen	5.8E-04
activation of protein kinase activity	5.8E-04
regulation of protein transport	5.8E-04
regulation of protein localization	5.8E-04
vesicular fraction	6.0E-04
cell cycle phase	6.0E-04
regulation of inflammatory response	6.6E-04
regulation of cyclin-dependent protein kinase ac	6.6E-04
T cell differentiation in the thymus	6.7E-04
T cell differentiation	6.9E-04
nucleolus	6.9E-04
regulation of synapse structure and activity	7.0E-04
positive regulation of nitric oxide biosynthetic pr	7.1E-04
T cell receptor signaling pathway	7.6E-04
regulation of secretion	7.7E-04
regulation of B cell activation	7.8E-04
regulation of muscle development	7.8E-04
leukocyte proliferation	7.9E-04
mononuclear cell proliferation	7.9E-04
positive regulation of phosphate metabolic proce	8.1E-04
positive regulation of phosphorus metabolic proc	8.1E-04
neuromuscular process	8.2E-04
regulation of T cell proliferation	8.2E-04

Protein kinase	8.4E-04
endomembrane system	8.6E-04
membrane invagination	8.7E-04
endocytosis	8.7E-04
22q12.2	8.7E-04
response to calcium ion	8.8E-04
positive regulation of lymphocyte proliferation	8.8E-04
negative regulation of transcription	8.8E-04
transcription factor binding	8.8E-04
positive regulation of NF-kappaB transcription fa	9.1E-04
regulation of production of molecular mediator o	9.1E-04
actin binding	9.2E-04
positive regulation of protein amino acid phosph	9.3E-04
Integrin signalling pathway	9.4E-04
epithelial cell differentiation	9.7E-04
regulation of isotype switching	9.8E-04
positive regulation of endothelial cell migration	9.8E-04
negative regulation of protein metabolic process	9.9E-04
12q24.31	9.9E-04
adenyl nucleotide binding	9.9E-04
