# Supplementary material for: Transcriptional changes associated with resistance to inhibitors of epidermal growth factor receptor revealed using metaanalysis
Source: BMC Cancer. 2015 May 7;15:369. doi: 10.1186/s12885-015-1337-3 (PMC4430867; doi:10.1186/s12885-015-1337-3)
Supplement: Additional file 4: — Ontological categories characteristically expressed in Erlotinib-sensitive vs. resistant cell lines. [file 12885_2015_1337_MOESM4_ESM.zip › 12885_2015_1337_add4.pdf]

Erlotinib: Overexpression in sensitive cells

Supplement 4

Term	p_Value
translation	5.6E-62
translational elongation	6.3E-45
structural constituent of ribosome	1.0E-43
cytosol	3.7E-41
ribosome	4.5E-41
3' -UTR-mediated translational regulation	3.8E-40
ribonucleoprotein complex	5.0E-40
Ribosome	4.6E-37
ribosomal subunit	9.9E-36
Protein biosynthesis	2.0E-33
cytosolic ribosome	2.7E-33
Gene Expression	8.4E-32
Metabolism of proteins	5.4E-29
Ribosomal protein	7.7E-28
cytosolic part	1.0E-26
RNA binding	2.8E-26
Influenza Infection	1.6E-25
ribosome biogenesis	6.6E-21
ribonucleoprotein complex biogenesis	1.6E-20
ncRNA metabolic process	4.7E-19
large ribosomal subunit	8.4E-19
structural molecule activity	1.3E-18
membrane-enclosed lumen	3.0E-18
small ribosomal subunit	1.1E-17
cytosolic large ribosomal subunit	6.7E-17
organelle lumen	1.9E-16
intracellular organelle lumen	2.0E-16
cytosolic small ribosomal subunit	3.3E-16
non-membrane-bounded organelle	4.4E-16
intracellular non-membrane-bounded organelle	4.4E-16
RNA processing	1.2E-15
Nucleic acid binding	2.9E-15
Protein metabolism and modification	5.2E-15
rRNA metabolic process	8.5E-15
ncRNA processing	4.6E-14
rRNA processing	9.4E-14
translation factor activity, nucleic acid binding	2.1E-11
mitochondrion	2.4E-10
nuclear lumen	3.0E-10
Translation factor	1.2E-09
nucleolus	1.2E-09
translation initiation factor activity	2.0E-09
Translation initiation factor	2.4E-09
Synthase and synthetase	3.4E-09
regulation of programmed cell death	3.3E-08
regulation of translation	3.7E-08
regulation of apoptosis	3.8E-08
regulation of cell death	4.0E-08
mitochondrial part	6.8E-08
ribosomal small subunit biogenesis	1.6E-07
ligase activity, forming aminoacyl-tRNA and rel	2.0E-07

ligase activity, forming carbon-oxygen bonds	2.0E-07
aminoacyl-tRNA ligase activity	2.0E-07
organelle envelope	2.0E-07
glycolysis	2.1E-07
envelope	2.3E-07
Translational regulation	2.9E-07
7q22.1	3.5E-07
mitochondrion organization	5.4E-07
Aminoacyl-tRNA synthetase	7.3E-07
7q11.23	7.8E-07
regulation of cellular protein metabolic process	7.9E-07
tRNA metabolic process	7.9E-07
tRNA aminoacylation for protein translation	9.5E-07
amino acid activation	9.5E-07
tRNA aminoacylation	9.5E-07
glucose catabolic process	9.9E-07
macromolecular complex assembly	1.1E-06
monosaccharide catabolic process	1.1E-06
mitochondrial matrix	1.1E-06
mitochondrial lumen	1.1E-06
rRNA metabolism	1.5E-06
ribosomal large subunit biogenesis	1.5E-06
Glycolysis	1.9E-06
pigment granule	2.2E-06
melanosome	2.2E-06
7p15	2.5E-06
macromolecular complex subunit organization	2.6E-06
Chaperone	2.8E-06
hexose catabolic process	3.1E-06
Metabolism of carbohydrates	3.3E-06
cell redox homeostasis	3.5E-06
Aminoacyl-tRNA biosynthesis	4.1E-06
nuclear envelope	4.5E-06
posttranscriptional regulation of gene expressio	4.8E-06
cellular carbohydrate catabolic process	5.1E-06
6q25.3	5.6E-06
cellular macromolecular complex subunit organ	5.7E-06
negative regulation of programmed cell death	6.7E-06
regulation of translational initiation	7.2E-06
protein complex assembly	7.2E-06
protein complex biogenesis	7.2E-06
negative regulation of cell death	7.3E-06
alcohol catabolic process	9.1E-06
negative regulation of apoptosis	9.6E-06
cellular macromolecular complex assembly	1.1E-05
tRNA binding	1.1E-05
endomembrane system	1.2E-05
generation of precursor metabolites and energy	1.6E-05
Amino acid activation	1.9E-05
translational initiation	2.2E-05
GTPase activity	2.7E-05
organellar ribosome	4.0E-05
mitochondrial ribosome	4.0E-05
cellular protein complex assembly	4.2E-05
anti-apoptosis	4.4E-05
protein targeting	4.5E-05

eukaryotic translation initiation factor 3 complex	5.3E-05
cell death	6.8E-05
enzyme binding	6.8E-05
carbohydrate catabolic process	7.5E-05
response to unfolded protein	7.7E-05
Synthetase	8.3E-05
intracellular protein transport	8.4E-05
Chaperonin	8.5E-05
death	8.7E-05
Glycolysis	9.5E-05
cell proliferation	9.8E-05
programmed cell death	1.0E-04
1p36.3-p36.2	1.1E-04
intracellular transport	1.2E-04
positive regulation of apoptosis	1.3E-04
cellular protein localization	1.4E-04
RNA helicase	1.4E-04
positive regulation of programmed cell death	1.5E-04
nucleotide binding	1.6E-04
cellular macromolecule localization	1.6E-04
Glycolysis / Gluconeogenesis	1.7E-04
positive regulation of cell death	1.7E-04
Cell cycle	1.8E-04
apoptosis	1.9E-04
regulation of cell proliferation	2.1E-04
rRNA binding	2.2E-04
tRNA metabolism	2.2E-04
kinase binding	2.3E-04
eukaryotic translation initiation factor 4F comple	2.4E-04
RNA splicing, via transesterification reactions	2.6E-04
nuclear mRNA splicing, via spliceosome	2.6E-04
RNA splicing, via transesterification reactions w	2.6E-04
7p22	2.6E-04
12q21.2	2.6E-04
Pathogenic Escherichia coli infection	3.2E-04
purine nucleotide binding	3.6E-04
identical protein binding	3.6E-04
purine ribonucleotide binding	3.8E-04
ribonucleotide binding	3.8E-04
22q13.1	3.8E-04
regulation of cell motion	3.8E-04
Synthase	3.9E-04
soluble fraction	4.0E-04
purine NTP-dependent helicase activity	4.2E-04
ATP-dependent helicase activity	4.2E-04
monosaccharide metabolic process	4.2E-04
Protein folding	4.4E-04
intramolecular oxidoreductase activity	4.4E-04
mRNA metabolic process	4.5E-04
protein folding	4.7E-04
response to protein stimulus	4.8E-04
regulation of cell cycle	4.9E-04
response to inorganic substance	4.9E-04
11p13	4.9E-04
proteasome complex	5.5E-04
negative regulation of cellular protein metabolic	6.2E-04

glucose metabolic process	6.4E-04
ribonucleoprotein complex assembly	6.7E-04
hexose metabolic process	7.8E-04
polyamine metabolic process	8.4E-04
organelle membrane	8.5E-04
mitochondrial envelope	9.0E-04
nucleoplasm	9.4E-04
cellular homeostasis	9.5E-04
chaperonin-containing T-complex	9.6E-04
helicase activity	9.7E-04

Overexpressed in resistant cells	
**Term**	**p_Value**
cytosol	3.0E-20
translational elongation	3.1E-18
structural constituent of ribosome	3.4E-18
ribosomal subunit	3.5E-17
cytosolic ribosome	2.4E-16
mitochondrion	1.5E-15
Ribosome	2.6E-14
ribosome	2.6E-14
Influenza Infection	4.2E-14
intracellular non-membrane-bounded organelle	8.2E-13
non-membrane-bounded organelle	8.2E-13
cytosolic part	9.7E-13
3' -UTR-mediated translational regulation	1.2E-12
translation	1.9E-12
organelle inner membrane	2.2E-12
mitochondrial inner membrane	7.1E-12
mitochondrial envelope	7.2E-12
Ribosomal protein	1.1E-10
mitochondrial membrane	1.4E-10
oxidative phosphorylation	1.5E-10
hydrogen ion transmembrane transporter activity	1.8E-10
mitochondrial part	3.1E-10
generation of precursor metabolites and energy	3.4E-10
Oxidoreductase	6.6E-10
ribonucleoprotein complex	1.9E-09
cytosolic small ribosomal subunit	2.2E-09
organelle envelope	2.7E-09
Metabolism of proteins	2.7E-09
envelope	3.1E-09
large ribosomal subunit	3.4E-09
organelle membrane	3.8E-09
Dehydrogenase	3.9E-09
Oxidative phosphorylation	4.2E-09
monovalent inorganic cation transmembrane tra	6.0E-09
structural molecule activity	1.3E-08
mitotic cell cycle	3.2E-08
small ribosomal subunit	3.3E-08
cytosolic large ribosomal subunit	6.5E-08
mitochondrial membrane part	8.2E-08
ATP synthesis coupled proton transport	1.3E-07
energy coupled proton transport, down electroch	1.3E-07
Protein biosynthesis	1.6E-07
Reductase	1.6E-07
oxidation reduction	2.7E-07
Diabetes pathways	5.4E-07
respiratory chain	8.0E-07
proton-transporting two-sector ATPase complex	8.6E-07
Parkinson's disease	1.3E-06
cell cycle	1.4E-06
Gene Expression	1.5E-06
Integration of energy metabolism	1.6E-06

membrane-enclosed lumen	2.2E-06
ion transmembrane transport	2.6E-06
Huntington's disease	4.2E-06
energy derivation by oxidation of organic compo	4.9E-06
cell cycle process	5.2E-06
Oxidative phosphorylation	5.3E-06
actin cytoskeleton	5.3E-06
intracellular organelle lumen	5.6E-06
11q13	5.6E-06
inorganic cation transmembrane transporter acti	5.6E-06
RNA binding	5.9E-06
17q25	6.7E-06
organelle lumen	6.8E-06
M phase of mitotic cell cycle	9.1E-06
proton transport	1.1E-05
cellular respiration	1.3E-05
mitosis	1.5E-05
nuclear division	1.5E-05
organelle fission	1.5E-05
hydrogen transport	1.7E-05
electron transport chain	1.8E-05
mitochondrial respiratory chain	2.4E-05
cell cycle phase	2.9E-05
regulation of apoptosis	3.4E-05
regulation of cell death	3.4E-05
cofactor binding	3.9E-05
Alzheimer's disease	4.1E-05
oxidoreductase activity, acting on NADH or NAD	4.2E-05
proton-transporting two-sector ATPase complex,	4.8E-05
mitochondrial proton-transporting ATP synthase	4.8E-05
regulation of programmed cell death	4.9E-05
Histone	5.1E-05
M phase	6.7E-05
vesicular fraction	7.6E-05
DNA packaging	8.4E-05
positive regulation of apoptosis	8.5E-05
cell division	9.1E-05
microsome	9.1E-05
nucleoside triphosphate biosynthetic process	9.5E-05
positive regulation of programmed cell death	1.0E-04
oxidoreductase activity, acting on NADH or NAD	1.1E-04
negative regulation of protein modification proce	1.1E-04
proton-transporting ATP synthase complex	1.1E-04
positive regulation of cell death	1.2E-04
ATP biosynthetic process	1.4E-04
Pathogenic Escherichia coli infection	1.4E-04
cytoskeleton	1.5E-04
proton-transporting ATPase activity, rotational m	1.6E-04
purine ribonucleoside triphosphate biosynthetic p	1.6E-04
mitochondrial lumen	1.9E-04
mitochondrial matrix	1.9E-04
ribonucleoside triphosphate biosynthetic process	1.9E-04
purine nucleoside triphosphate biosynthetic proc	1.9E-04
negative regulation of molecular function	1.9E-04
Hydrogen transporter	2.0E-04
oxidoreductase activity, acting on diphenols and	2.2E-04

NADH dehydrogenase (ubiquinone) activity	2.3E-04
NADH dehydrogenase activity	2.3E-04
NADH dehydrogenase (quinone) activity	2.3E-04
mitochondrial ATP synthesis coupled electron tra	2.8E-04
ATP synthesis coupled electron transport	2.8E-04
nucleosome	3.0E-04
protein-DNA complex	3.1E-04
vacuole	3.2E-04
apicolateral plasma membrane	3.2E-04
respiratory electron transport chain	3.2E-04
epithelium development	3.4E-04
Cell Cycle, Mitotic	3.4E-04
programmed cell death	3.7E-04
anaphase-promoting complex-dependent protea	3.8E-04
negative regulation of ubiquitin-protein ligase ac	3.8E-04
apoptosis	4.0E-04
response to organic substance	4.2E-04
phosphorus metabolic process	4.2E-04
phosphate metabolic process	4.2E-04
nucleotide binding	4.5E-04
Electron transport	4.7E-04
negative regulation of protein ubiquitination	4.8E-04
negative regulation of ubiquitin-protein ligase ac	5.3E-04
negative regulation of ligase activity	5.3E-04
apical junction complex	6.2E-04
positive regulation of ubiquitin-protein ligase act	6.2E-04
purine ribonucleoside triphosphate metabolic pro	6.4E-04
ribonucleoprotein binding	6.5E-04
identical protein binding	6.7E-04
response to virus	6.7E-04
14q11.2	6.8E-04
negative regulation of cellular protein metabolic	7.0E-04
hydrogen ion transporting ATP synthase activity	7.0E-04
ribonucleoside triphosphate metabolic process	7.1E-04
11q23.3	7.2E-04
Protein metabolism and modification	7.4E-04
proteasomal protein catabolic process	7.9E-04
proteasomal ubiquitin-dependent protein catabol	7.9E-04
positive regulation of ubiquitin-protein ligase act	8.4E-04
coenzyme binding	8.9E-04
nucleobase, nucleoside and nucleotide biosynthe	9.5E-04
nucleobase, nucleoside, nucleotide and nucleic a	9.5E-04
chromatin assembly	9.6E-04
regulation of ubiquitin-protein ligase activity duri	9.7E-04
ATPase activity, coupled to transmembrane mov	9.8E-04
