# Supplementary material for: Transcriptional changes associated with resistance to inhibitors of epidermal growth factor receptor revealed using metaanalysis
Source: BMC Cancer. 2015 May 7;15:369. doi: 10.1186/s12885-015-1337-3 (PMC4430867; doi:10.1186/s12885-015-1337-3)
Supplement: Additional file 5: — Ontological categories characteristically expressed in Gefitinib-sensitive vs. resistant cell lines. [file 12885_2015_1337_MOESM5_ESM.zip › 12885_2015_1337_add5.pdf]

Gefitinib: Overexpressed in sensitive cells

Supplement 5

**Term**	**p-Value**
Pathways in cancer	6.8E-14
Pancreatic cancer	1.2E-07
protein complex assembly	3.7E-06
protein complex biogenesis	3.7E-06
regulation of cell proliferation	3.9E-06
Bladder cancer	7.8E-06
regulation of protein kinase cascade	8.7E-06
cell adhesion	9.0E-06
biological adhesion	9.8E-06
positive regulation of macromolecule metabolic	1.0E-05
Colorectal cancer	1.4E-05
positive regulation of developmental process	1.6E-05
cell proliferation	1.6E-05
regulation of phosphorus metabolic process	2.1E-05
regulation of phosphate metabolic process	2.1E-05
placenta development	2.3E-05
regulation of phosphorylation	2.3E-05
macromolecular complex subunit organization	2.4E-05
in utero embryonic development	2.4E-05
regulation of transcription from RNA polymeras	2.6E-05
forebrain development	2.6E-05
regulation of protein amino acid phosphorylatio	2.7E-05
regulation of lipid metabolic process	3.1E-05
intracellular signaling cascade	3.3E-05
regulation of cell death	3.6E-05
10q24	3.7E-05
endomembrane system	3.7E-05
homeostatic process	3.7E-05
regulation of programmed cell death	4.0E-05
Chronic myeloid leukemia	4.2E-05
negative regulation of cell death	4.4E-05
Leukocyte transendothelial migration	6.2E-05
negative regulation of programmed cell death	6.3E-05
cell migration	6.3E-05
macromolecular complex assembly	6.5E-05
positive regulation of cell proliferation	7.3E-05
Intracellular signaling cascade	7.6E-05
positive regulation of protein amino acid phospl	7.9E-05
regulation of apoptosis	7.9E-05
chordate embryonic development	8.7E-05
establishment of protein localization	8.8E-05
positive regulation of molecular function	1.0E-04
positive regulation of transcription from RNA pc	1.1E-04
anti-apoptosis	1.1E-04
protein localization	1.1E-04
cellular cation homeostasis	1.2E-04
embryonic development ending in birth or egg h	1.3E-04
positive regulation of protein kinase cascade	1.3E-04
positive regulation of cell differentiation	1.3E-04
negative regulation of apoptosis	1.4E-04

3p21.3	1.5E-04
positive regulation of macromolecule biosynthe	1.5E-04
5q31-q32	1.6E-04
kinase binding	1.6E-04
purine nucleotide binding	1.7E-04
chemical homeostasis	1.7E-04
cellular di-, tri-valent inorganic cation homeosta	1.7E-04
nucleotide binding	1.8E-04
positive regulation of signal transduction	1.9E-04
protein transport	1.9E-04
regulation of transferase activity	1.9E-04
activation of pro-apoptotic gene products	2.0E-04
regulation of cellular protein metabolic process	2.0E-04
purine nucleoside binding	2.2E-04
positive regulation of biosynthetic process	2.2E-04
adenyl nucleotide binding	2.3E-04
3q21	2.3E-04
cellular component morphogenesis	2.3E-04
positive regulation of phosphate metabolic proc	2.4E-04
positive regulation of phosphorus metabolic pro	2.4E-04
Neurotrophin signaling pathway	2.5E-04
regulation of I-kappaB kinase/NF-kappaB casca	2.5E-04
nucleolus	2.5E-04
nucleoside binding	2.5E-04
positive regulation of phosphorylation	2.6E-04
perinuclear region of cytoplasm	2.7E-04
positive regulation of cell communication	2.9E-04
blastocyst formation	2.9E-04
cell motility	3.2E-04
localization of cell	3.2E-04
cell motion	3.2E-04
positive regulation of lipid metabolic process	3.4E-04
positive regulation of cellular biosynthetic proce	3.6E-04
Prostate cancer	3.8E-04
transcription activator activity	4.0E-04
regulation of locomotion	4.2E-04
Metabolism of vitamins and cofactors	4.3E-04
Focal adhesion	4.4E-04
purine ribonucleotide binding	4.6E-04
ribonucleotide binding	4.6E-04
regulation of protein modification process	4.9E-04
positive regulation of nitrogen compound metab	4.9E-04
plasma membrane part	4.9E-04
regulation of kinase activity	4.9E-04
nuclear lumen	5.1E-04
Acute myeloid leukemia	5.2E-04
ATP binding	5.2E-04
11p15.3	5.2E-04
ErbB signaling pathway	5.2E-04
Endometrial cancer	5.5E-04
cell fraction	5.7E-04
Melanoma	5.7E-04
di-, tri-valent inorganic cation homeostasis	5.7E-04
cell projection	6.2E-04
T cell receptor signaling pathway	6.4E-04
cellular homeostasis	6.4E-04

identical protein binding	6.6E-04
enzyme binding	6.7E-04
response to ethanol	6.9E-04
negative regulation of macromolecule metaboli	6.9E-04
positive regulation of nucleobase, nucleoside, n	6.9E-04
negative regulation of phosphate metabolic pro	7.0E-04
negative regulation of phosphorus metabolic pr	7.0E-04
negative regulation of phosphorylation	7.3E-04
regulation of cytokine production	7.5E-04
adenyl ribonucleotide binding	7.6E-04
16q22.1	7.7E-04
regulation of peptidyl-tyrosine phosphorylation	8.3E-04
17p13.2	8.9E-04
telencephalon development	9.1E-04
regulation of binding	9.5E-04
negative regulation of macromolecule biosynthe	9.6E-04
11p15.5	9.6E-04
regulation of T cell activation	9.7E-04
endoplasmic reticulum	9.8E-04
regulation of T cell proliferation	9.8E-04
Oncogenesis	9.9E-04

Overexpressed in resistant cells	
**Term**	**p-Value**
positive regulation of biosynthetic process	1.7E-11
positive regulation of macromolecule metabolic	3.1E-11
positive regulation of cellular biosynthetic proces	3.5E-11
positive regulation of nitrogen compound metab	1.6E-10
positive regulation of macromolecule biosynthet	4.4E-10
response to endogenous stimulus	5.1E-10
positive regulation of nucleobase, nucleoside, nu	3.6E-09
response to hormone stimulus	8.1E-09
Pathways in cancer	1.0E-08
positive regulation of transcription	1.5E-08
positive regulation of gene expression	2.1E-08
regulation of cell motion	2.5E-08
regulation of cell proliferation	3.5E-08
response to steroid hormone stimulus	4.5E-08
regulation of locomotion	4.5E-08
hemopoietic or lymphoid organ development	6.3E-08
response to organic substance	7.3E-08
regulation of cell migration	9.8E-08
hemopoiesis	1.2E-07
regulation of phosphorylation	1.5E-07
cell activation	2.3E-07
positive regulation of transcription, DNA-depend	2.5E-07
positive regulation of RNA metabolic process	2.6E-07
positive regulation of cell migration	3.2E-07
positive regulation of locomotion	3.3E-07
positive regulation of cell motion	3.3E-07
response to nutrient levels	3.6E-07
transmembrane receptor protein tyrosine kinase	3.7E-07
immune system development	4.1E-07
Thyroid cancer	6.2E-07
response to extracellular stimulus	6.8E-07
intracellular signaling cascade	6.9E-07
negative regulation of apoptosis	8.1E-07
regulation of cytokine production	9.2E-07
leukocyte activation	9.2E-07
negative regulation of programmed cell death	1.0E-06
negative regulation of cell death	1.2E-06
regulation of phosphate metabolic process	1.3E-06
regulation of phosphorus metabolic process	1.3E-06
regulation of neuron apoptosis	1.4E-06
regulation of protein amino acid phosphorylation	1.5E-06
positive regulation of cell proliferation	1.9E-06
positive regulation of molecular function	2.1E-06
response to drug	2.5E-06
extracellular region part	2.8E-06
regulation of cytokine biosynthetic process	2.8E-06
regulation of cellular protein metabolic process	3.6E-06
positive regulation of developmental process	4.1E-06
negative regulation of transport	4.9E-06
cytoskeleton organization	5.3E-06

anti-apoptosis	5.4E-06
regulation of response to external stimulus	6.1E-06
regulation of programmed cell death	6.5E-06
regulation of protein modification process	6.6E-06
Adherens junction	7.0E-06
regulation of cell death	8.6E-06
response to hypoxia	8.7E-06
response to estrogen stimulus	8.9E-06
regulation of apoptosis	9.1E-06
leukocyte differentiation	9.4E-06
response to oxygen levels	9.7E-06
regulation of cell activation	1.1E-05
positive regulation of transcription factor activity	1.2E-05
enzyme binding	1.4E-05
cell-cell signaling	1.6E-05
positive regulation of DNA binding	1.8E-05
response to peptide hormone stimulus	1.9E-05
regulation of leukocyte activation	2.9E-05
regulation of transcription from RNA polymerase	3.1E-05
response to insulin stimulus	3.3E-05
response to inorganic substance	3.4E-05
positive regulation of binding	3.4E-05
response to nutrient	3.7E-05
response to vitamin	3.7E-05
protein kinase cascade	4.3E-05
response to corticosteroid stimulus	4.3E-05
regulation of lymphocyte activation	4.9E-05
blood circulation	4.9E-05
circulatory system process	4.9E-05
enzyme linked receptor protein signaling pathwa	5.4E-05
Prostate cancer	5.7E-05
negative regulation of macromolecule metabolic	5.9E-05
cell motion	6.3E-05
Focal adhesion	7.0E-05
response to wounding	7.3E-05
lymphocyte differentiation	7.4E-05
tissue remodeling	7.8E-05
regulation of growth	7.8E-05
positive regulation of transcription from RNA pol	7.9E-05
regulation of cell adhesion	8.5E-05
regulation of transferase activity	8.9E-05
regulation of T cell activation	9.0E-05
response to glucocorticoid stimulus	9.1E-05
identical protein binding	9.8E-05
Bladder cancer	1.0E-04
regulation of angiogenesis	1.1E-04
positive regulation of cytokine biosynthetic proce	1.1E-04
negative regulation of neuron apoptosis	1.1E-04
11p15.5	1.1E-04
response to estradiol stimulus	1.1E-04
blood vessel development	1.2E-04
Signaling in Immune system	1.3E-04
regulation of protein kinase activity	1.3E-04
regulation of protein kinase cascade	1.3E-04
regulation of kinase activity	1.4E-04
response to molecule of bacterial origin	1.4E-04

promoter binding	1.6E-04
response to lipopolysaccharide	1.7E-04
vasculature development	1.8E-04
regulation of blood pressure	1.8E-04
Small cell lung cancer	1.9E-04
Colorectal cancer	1.9E-04
regulation of epithelial cell proliferation	1.9E-04
cofactor biosynthetic process	2.0E-04
cell morphogenesis	2.0E-04
cellular response to insulin stimulus	2.0E-04
protein tyrosine kinase activity	2.1E-04
Endometrial cancer	2.1E-04
cell fraction	2.3E-04
cell cycle process	2.3E-04
nuclear lumen	2.4E-04
response to reactive oxygen species	2.4E-04
extracellular space	2.5E-04
cell projection	2.5E-04
positive regulation of cellular component organiz	2.5E-04
lymphocyte activation	2.6E-04
cell cycle	2.7E-04
membrane-enclosed lumen	2.8E-04
regulation of lipid metabolic process	2.9E-04
cytosol	2.9E-04
positive regulation of cell communication	2.9E-04
positive regulation of cell differentiation	3.0E-04
Intracellular signaling cascade	3.1E-04
positive regulation of multicellular organismal pr	3.2E-04
response to abiotic stimulus	3.4E-04
negative regulation of phosphorylation	3.4E-04
Viral myocarditis	3.5E-04
organelle lumen	3.5E-04
aging	3.9E-04
positive regulation of catalytic activity	4.0E-04
positive regulation of kinase activity	4.0E-04
positive regulation of response to external stimu	4.1E-04
cellular component morphogenesis	4.3E-04
positive regulation of phosphorylation	4.4E-04
vesicle-mediated transport	4.4E-04
positive regulation of signal transduction	4.6E-04
regulation of mononuclear cell proliferation	4.8E-04
regulation of leukocyte proliferation	4.8E-04
regulation of oxidoreductase activity	4.9E-04
myeloid leukocyte activation	4.9E-04
glucose metabolic process	5.1E-04
regulation of binding	5.1E-04
negative regulation of multicellular organismal p	5.4E-04
monosaccharide metabolic process	5.4E-04
skeletal system development	5.4E-04
chromatin modification	5.5E-04
regulation of immunoglobulin production	5.5E-04
regulation of cellular component size	6.0E-04
heterocycle biosynthetic process	6.1E-04
cell projection morphogenesis	6.1E-04
positive regulation of protein kinase activity	6.2E-04
response to hydrogen peroxide	6.3E-04

negative regulation of cell differentiation	6.6E-04
regulation of striated muscle cell differentiation	6.7E-04
negative regulation of response to external stimu	6.8E-04
intracellular organelle lumen	6.9E-04
inflammatory response	7.0E-04
response to cytokine stimulus	7.1E-04
cell-matrix adhesion	7.1E-04
positive regulation of protein amino acid phosph	7.1E-04
cofactor metabolic process	7.6E-04
regulation of cell size	7.6E-04
response to organic nitrogen	7.7E-04
localization of cell	7.8E-04
cell motility	7.8E-04
regulation of transcription factor activity	7.9E-04
transcription, DNA-dependent	7.9E-04
regulation of synapse structure and activity	8.1E-04
positive regulation of response to stimulus	8.1E-04
Non-receptor tyrosine protein kinase	8.1E-04
negative regulation of growth	8.1E-04
RNA biosynthetic process	8.2E-04
neuron differentiation	8.2E-04
regulation of lymphocyte proliferation	8.4E-04
positive regulation of transferase activity	8.4E-04
axon	8.5E-04
positive regulation of phosphate metabolic proce	8.6E-04
positive regulation of phosphorus metabolic proc	8.6E-04
Extracellular matrix protein-mediated signaling	8.8E-04
positive regulation of cellular protein metabolic p	8.9E-04
regulation of cell cycle	9.0E-04
positive regulation of cytokine production	9.0E-04
regulation of cellular localization	9.1E-04
Allograft rejection	9.1E-04
regulation of cyclin-dependent protein kinase ac	9.1E-04
regulation of nitric oxide biosynthetic process	9.1E-04
positive regulation of growth	9.1E-04
regulation of DNA binding	9.1E-04
muscle cell differentiation	9.1E-04
blood vessel morphogenesis	9.2E-04
Kinase modulator	9.2E-04
positive regulation of lymphocyte activation	9.5E-04
neuron projection development	9.7E-04
cell part morphogenesis	9.7E-04
cell proliferation	9.8E-04
negative regulation of phosphorus metabolic pro	9.9E-04
negative regulation of phosphate metabolic proc	9.9E-04
positive regulation of cell activation	1.0E-03
