# Supplementary material for: Transcriptional changes associated with resistance to inhibitors of epidermal growth factor receptor revealed using metaanalysis
Source: BMC Cancer. 2015 May 7;15:369. doi: 10.1186/s12885-015-1337-3 (PMC4430867; doi:10.1186/s12885-015-1337-3)
Supplement: Additional file 6: — Ontological categories characteristically expressed in irreversible inhibitors-sensitive vs. resistant cell lines. [file 12885_2015_1337_MOESM6_ESM.zip › 12885_2015_1337_add6.pdf]

Irreversible: Overexpressed in sensitive cells	
**Supplement 6**	
**Term**	**p_Value**
endomembrane system	3.8E-09
Golgi apparatus	2.4E-07
cell fraction	1.0E-05
cell division	2.1E-05
organelle membrane	3.1E-05
nuclear envelope-endoplasmic reticulum netwo	5.9E-05
response to organic substance	8.1E-05
endoplasmic reticulum membrane	1.0E-04
Mitosis	1.1E-04
1p22.1	1.5E-04
endoplasmic reticulum	1.9E-04
nuclear envelope	2.5E-04
cell cycle	3.1E-04
steroid metabolic process	4.3E-04
1p22	4.8E-04
endoplasmic reticulum part	6.3E-04
membrane fraction	8.0E-04
Select regulatory molecule	9.5E-04

Overexpressed in resistant cells	
**Term**	**p_Value**
extracellular region	1.6E-08
ectoderm development	3.3E-07
response to wounding	4.3E-07
extracellular region part	1.3E-06
extracellular space	2.4E-06
epidermis development	8.4E-06
regulation of cell proliferation	1.1E-05
regulation of smooth muscle cell proliferation	6.5E-05
wound healing	7.3E-05
positive regulation of smooth muscle cell prolifer	1.9E-04
epidermal growth factor receptor binding	2.5E-04
apical plasma membrane	3.1E-04
Structural protein	3.3E-04
apical part of cell	3.5E-04
plasma membrane part	3.6E-04
Signaling molecule	4.2E-04
ErbB signaling pathway	4.2E-04
positive regulation of cell proliferation	4.8E-04
intrinsic to plasma membrane	5.6E-04
defense response	5.9E-04
inflammatory response	9.2E-04
integral to plasma membrane	9.8E-04
