# Supplementary material for: Transcriptional changes associated with resistance to inhibitors of epidermal growth factor receptor revealed using metaanalysis
Source: BMC Cancer. 2015 May 7;15:369. doi: 10.1186/s12885-015-1337-3 (PMC4430867; doi:10.1186/s12885-015-1337-3)
Supplement: Additional file 7: — Ontological categories characteristically expressed in Cetuximab-sensitive vs. resistant cell lines. [file 12885_2015_1337_MOESM7_ESM.zip › 12885_2015_1337_add7.pdf]

Cetuximab: Overexpressed in sensitive cells	
**Supplement 7**	
**Term**	**P_value**
response to wounding	1.3E-10
regulation of cell proliferation	1.6E-09
positive regulation of cell proliferation	3.5E-07
chemotaxis	8.9E-07
taxis	8.9E-07
cell migration	1.7E-06
localization of cell	2.6E-06
cell motility	2.6E-06
Cell communication	3.5E-06
regulation of response to external stimulus	4.0E-06
Ligand-mediated signaling	4.6E-06
cell motion	5.3E-06
inflammatory response	6.5E-06
wound healing	9.1E-06
immune response	9.8E-06
regulation of protein amino acid phosphorylatio	1.1E-05
Signal transduction	1.5E-05
positive regulation of locomotion	1.5E-05
Intracellular signaling cascade	2.4E-05
homeostatic process	2.5E-05
regulation of vascular endothelial growth factor	3.2E-05
growth factor binding	3.3E-05
positive regulation of response to stimulus	3.4E-05
Cytokine and chemokine mediated signaling pa	3.7E-05
angiogenesis	3.8E-05
extracellular region part	4.0E-05
Signaling molecule	5.0E-05
Cytokine-cytokine receptor interaction	5.9E-05
positive regulation of smooth muscle cell prolife	6.5E-05
Immunity and defense	6.7E-05
regulation of secretion	7.0E-05
regulation of smooth muscle cell proliferation	8.0E-05
positive regulation of developmental process	8.2E-05
positive regulation of cell motion	8.6E-05
behavior	9.0E-05
negative regulation of response to stimulus	1.0E-04
blood vessel morphogenesis	1.1E-04
chemical homeostasis	1.4E-04
cell adhesion	1.8E-04
biological adhesion	1.8E-04
blood vessel development	1.8E-04
cytokine activity	1.9E-04
cytokine-mediated signaling pathway	2.0E-04
extracellular space	2.0E-04
positive regulation of cell migration	2.1E-04
defense response	2.1E-04
regulation of cellular localization	2.1E-04
ion homeostasis	2.1E-04
locomotory behavior	2.1E-04
vasculature development	2.4E-04
cell chemotaxis	2.5E-04

regulation of inflammatory response	3.5E-04
cell activation	3.7E-04
regulation of body fluid levels	4.2E-04
positive regulation of immune system process	4.3E-04
cellular ion homeostasis	4.4E-04
positive regulation of phosphorus metabolic pro	5.0E-04
positive regulation of phosphate metabolic proc	5.0E-04
regulation of protein modification process	5.1E-04
Jak-STAT signaling pathway	5.1E-04
cellular chemical homeostasis	5.3E-04
positive regulation of immune response	5.4E-04
blood coagulation	5.8E-04
coagulation	5.8E-04
negative regulation of signal transduction	6.2E-04
positive regulation vascular endothelial growth f	6.2E-04
negative regulation of cell communication	6.5E-04
positive regulation of response to external stimu	6.5E-04
neutrophil chemotaxis	6.6E-04
JAK-STAT cascade	6.7E-04
negative regulation of cell proliferation	7.3E-04
Hematopoietic cell lineage	8.0E-04
hemostasis	8.7E-04
12q14.2	8.7E-04
regulation of phosphate metabolic process	8.7E-04
regulation of phosphorus metabolic process	8.7E-04
positive regulation of transcription from RNA pc	1.0E-03

Overexpressed in resistant cells	
**Term**	**p_Value**
ectoderm development	9.2E-10
epidermis development	1.4E-09
epithelium development	1.0E-08
epithelial cell differentiation	1.5E-07
keratinocyte differentiation	6.2E-05
cornified envelope	6.5E-05
extracellular matrix	7.8E-05
epidermal cell differentiation	1.2E-04
peptide cross-linking	1.7E-04
wound healing	1.7E-04
proteinaceous extracellular matrix	2.8E-04
Cell structure and motility	3.3E-04
regulation of cell proliferation	4.3E-04
Ectoderm development	5.6E-04
12q12-q13	6.9E-04
9q34.3	7.2E-04
anchoring junction	7.6E-04
transcription repressor activity	8.4E-04
