# Supplementary material for: Transcriptional changes associated with resistance to inhibitors of epidermal growth factor receptor revealed using metaanalysis
Source: BMC Cancer. 2015 May 7;15:369. doi: 10.1186/s12885-015-1337-3 (PMC4430867; doi:10.1186/s12885-015-1337-3)
Supplement: Additional file 8: — a. Cluster analysis for Erlotinib Sensitivevs.regulated analysis using DAVID software. For complete lists see Additional file 4. Additional files 8b and 8c Clusters of differentially expressed gene ontologies in irreversible inhibitor- and Cetuximab-sensitive vs. resistant cell lines, respectively. For complete lists see Additional files 6 and 7. [file 12885_2015_1337_MOESM8_ESM.zip › 12885_2015_1337_add8a.pdf]

9a	Erlotinib			
	Sensitive			Resistant
T	**Enrichment Score: 31.29**	**p_Value**	T	**Enrichment Score: 11.14**
	translation	5.6E-62		translational elongation
	Protein biosynthesis	2.0E-33		Metabolism of proteins
T	**Enrichment Score: 16.23**			**Enrichment Score: 9.32**
	ribosome biogenesis	6.6E-21		non-membrane-bounded organelle
	RNA processing	1.2E-15	CY	cytoskeleton
T	**Enrichment Score: 11.73**		E	**Enrichment Score: 8.05**
	membrane-enclosed lumen	3.0E-18		Oxidoreductase
	nucleolus	1.2E-09		Dehydrogenase
T	**Enrichment Score: 7.19**		E	**Enrichment Score: 6.26**
	translation factor activity, nucleic ac	2.1E-11		mitochondrion
	regulation of translational initiation	7.2E-06		oxidative phosphorylation
T	**Enrichment Score: 6.08**		E	**Enrichment Score: 5.65**
	Synthase and synthetase	3.4E-09		mitochondrial part
	tRNA aminoacylation for protein trar	9.5E-07		mitochondrial lumen
	**Enrichment Score: 6.00**		CC	**Enrichment Score: 5.08**
	regulation of translation	3.7E-08		mitotic cell cycle
	regulation of cellular protein metabo	7.9E-07		M phase of mitotic cell cycle
T	**Enrichment Score: 5.20**		T	**Enrichment Score: 3.68**
	macromolecular complex assembly	1.1E-06		membrane-enclosed lumen
	cellular protein complex assembly	4.2E-05		nuclear lumen
A	**Enrichment Score: 4.62**		A	**Enrichment Score: 3.27**
	regulation of programmed cell deatr	3.3E-08		regulation of apoptosis
	negative regulation of programmed	6.7E-06		positive regulation of apoptosis
	positive regulation of apoptosis	1.3E-04		negative regulation of cell death
M_c	**Enrichment Score: 4.55**		MM	**Enrichment Score: 2.87**
	glycolysis	2.1E-07		vesicular fraction
	Glycolysis / Gluconeogenesis	1.7E-04		microsome
E	**Enrichment Score: 4.54**		CY	**Enrichment Score: 2.79**
	mitochondrion	2.4E-10		cytoskeleton
	envelope	2.3E-07		microtubule cytoskeleton
A	**Enrichment Score: 3.98**		CY	**Enrichment Score: 2.76**
	cell death	6.8E-05		actin cytoskeleton
	apoptosis	1.9E-04		actin binding
T	**Enrichment Score: 3.69**			Actin binding cytoskeletal protein
	Chaperone	2.8E-06	MM	**Enrichment Score: 2.76**
	unfolded protein binding	2.8E-03		vacuole
T	**Enrichment Score: 3.43**			Lysosome
	mitochondrial lumen	1.1E-06	E	**Enrichment Score: 2.67**
	mitochondrial ribosome	4.0E-05		oxidative phosphorylation
MM	**Enrichment Score: 3.22**			ATP biosynthetic process
	melanosome	2.2E-06	CY	**Enrichment Score: 2.54**
	cytoplasmic membrane-bounded ve	1.4E-02		cytoskeleton organization
T	**Enrichment Score: 3.05**			actin cytoskeleton organization
	protein targeting	4.5E-05	S	**Enrichment Score: 2.29**
	intracellular protein transport	8.4E-05		protein binding, bridging
MO	**Enrichment Score: 2.90**			molecular adaptor activity
	regulation of cell motion	3.8E-04	T	**Enrichment Score: 2.25**
	positive regulation of cell migration	3.1E-03		negative regulation of protein modific
S	**Enrichment Score: 2.65**			negative regulation of protein ubiquiti
	nucleotide binding	1.6E-04		positive regulation of ubiquitin-proteir
	ATP binding	7.1E-03	S	**Enrichment Score: 2.22**

T	**Enrichment Score: 2.65**	
	Chaperonin	8.5E-05
	Protein complex assembly	1.1E-01
T	**Enrichment Score: 2.56**	
	RNA helicase	1.4E-04
	ATPase activity, coupled	6.8E-02
R	**Enrichment Score: 2.46**	
	response to inorganic substance	4.9E-04
	response to calcium ion	8.5E-03
T	**Enrichment Score: 2.40**	
	protein targeting	4.5E-05
	protein import into nucleus, docking	7.6E-03
T	**Enrichment Score: 2.40**	
	RNA splicing, via transesterification	2.6E-04
	nuclear mRNA splicing, via spliceos	2.6E-04
V	**Enrichment Score: 2.36**	
	blood vessel morphogenesis	1.6E-03
	angiogenesis	4.7E-03
E	**Enrichment Score: 2.34**	
	generation of precursor metabolites	1.6E-05
	Integration of energy metabolism	8.7E-02
S	**Enrichment Score: 2.25**	
	GTPase activity	2.7E-05
	G Protein	1.1E-02
E	**Enrichment Score: 2.20**	
	protein disulfide oxidoreductase acti	2.9E-03
	oxidoreductase activity, acting on su	8.5E-03
M_c	**Enrichment Score: 2.18**	
	Glycolysis	9.5E-05
	Fructose and mannose metabolism	4.6E-02
	Fructose galactose metabolism	6.2E-02

	nucleotide binding
	ATP binding
MM	**Enrichment Score: 2.14**
	melanosome
	cytoplasmic membrane-bounded ves
CC	**Enrichment Score: 2.09**
	Histone
	DNA packaging
CC	**Enrichment Score: 2.08**
	microtubule cytoskeleton
	microtubule organizing center
AD	**Enrichment Score: 2.03**
	apicolateral plasma membrane
	apical junction complex

**p_Value**
3.1E-18
2.7E-09
8.2E-13
1.5E-04
6.6E-10
3.9E-09
1.5E-15
1.5E-10
3.1E-10
1.9E-04
3.2E-08
9.1E-06
2.2E-06
2.5E-03
3.4E-05
8.5E-05
3.0E-03
7.6E-05
9.1E-05
1.5E-04
1.3E-03
5.3E-06
2.9E-02
3.2E-02
3.2E-04
1.7E-03
1.5E-10
1.4E-04
1.2E-03
5.0E-03
1.4E-03
1.0E-02
1.1E-04
4.8E-04
8.4E-04

4.5E-04
1.3E-02

1.4E-03
1.9E-01

5.1E-05
8.4E-05

1.3E-03
2.5E-02

3.2E-04
6.2E-04
