# Supplementary material for: Transcriptional changes associated with resistance to inhibitors of epidermal growth factor receptor revealed using metaanalysis
Source: BMC Cancer. 2015 May 7;15:369. doi: 10.1186/s12885-015-1337-3 (PMC4430867; doi:10.1186/s12885-015-1337-3)
Supplement: Additional file 8: — a. Cluster analysis for Erlotinib Sensitivevs.regulated analysis using DAVID software. For complete lists see Additional file 4. Additional files 8b and 8c Clusters of differentially expressed gene ontologies in irreversible inhibitor- and Cetuximab-sensitive vs. resistant cell lines, respectively. For complete lists see Additional files 6 and 7. [file 12885_2015_1337_MOESM8_ESM.zip › 12885_2015_1337_add8bc.pdf]

9b	Irreversible inhibitors				
	Sensitive			Resistant	
MM	**Enrichment Score: 4.67**	**p_Value**	EC	**Enrichment Score: 6.42**	**p_Value**
	endomembrane system	3.8E-09		extracellular region	1.6E-08
	nuclear envelope-endoplasmic retic	5.9E-05		extracellular space	2.4E-06
MM	**Enrichment Score: 3.08**		IM	**Enrichment Score: 2.44**	
	cell fraction	1.0E-05		response to wounding	4.3E-07
	microsome	8.3E-03		inflammatory response	9.2E-04
R	**Enrichment Score: 2.60**		V	**Enrichment Score: 2.29**	
	response to organic substance	8.1E-05		angiogenesis	2.6E-03
	response to hormone stimulus	2.8E-02		vasculature development	9.1E-03
CC	**Enrichment Score: 2.42**		S	**Enrichment Score: 2.29**	
	cell division	2.1E-05		epidermal growth factor receptor binc	2.5E-04
	M phase of mitotic cell cycle	9.3E-03		regulation of epidermal growth factor	1.9E-02
			M	**Enrichment Score: 2.26**	
				regulation of locomotion	2.1E-03
				positive regulation of cell migration	9.5E-03
				**Enrichment Score: 2.13**	
			MM	epidermis development	8.4E-06
			DF	keratinocyte differentiation	2.4E-02
			MM	**Enrichment Score: 2.01**	
				plasma membrane part	3.6E-04
				integral to plasma membrane	9.8E-04
				**Enrichment Score: 2.00**	
			MM	ectoderm development	3.3E-07
			CY	Intermediate filament	7.8E-03

9c	Cetuximab				
	Sensitive			Resistant	
IM	**Enrichment Score: 6.25**	**PValue**	DF	**Enrichment Score: 5.26**	**PValue**
	response to wounding	1.3E-10		ectoderm development	9.2E-10
	inflammatory response	6.5E-06		keratinocyte differentiation	6.2E-05
M	**Enrichment Score: 5.25**		EC	**Enrichment Score: 2.24**	
	cell migration	1.7E-06		extracellular matrix	7.8E-05
	localization of cell	2.6E-06		proteinaceous extracellular matrix	2.8E-04
M	**Enrichment Score: 4.68**		AD	**Enrichment Score: 2.12**	
	taxis	8.9E-07		anchoring junction	7.6E-04
	cell chemotaxis	2.5E-04		cell-substrate adherens junction	2.1E-02
IM	**Enrichment Score: 4.00**				
	regulation of response to external st	4.0E-06			
	regulation of inflammatory response	3.5E-04			
S	**Enrichment Score: 3.98**				
	Cell communication	3.5E-06			
	Cell surface receptor mediated signa	4.3E-02			
V	**Enrichment Score: 3.92**				
	angiogenesis	3.8E-05			
	vasculature development	2.4E-04			
M	**Enrichment Score: 3.38**				
	positive regulation of locomotion	1.5E-05			
	positive regulation of cell migration	2.1E-04			
AD	**Enrichment Score: 3.02**				
	cell adhesion	1.8E-04			
	cell-cell adhesion	2.7E-02			
IM	**Enrichment Score: 2.88**				
	wound healing	9.1E-06			

I

	blood coagulation	5.8E-04		
A	**Enrichment Score: 2.67**			
	Jak-STAT signaling pathway	5.1E-04		
	Inhibition of apoptosis	2.8E-02		
H	**Enrichment Score: 2.65**			
	homeostatic process	2.5E-05		
	cytosolic calcium ion homeostasis	6.0E-03		
IM	**Enrichment Score: 2.55**			
	negative regulation of response to s	1.0E-04		
	negative regulation of defense respo	1.3E-03		
IM	**Enrichment Score: 2.19**			
	positive regulation of response to ex	6.5E-04		
	positive regulation of defense respo	1.4E-03		
	**Enrichment Score: 2.18**			
	regulation of vascular endothelial gr	3.2E-05		
	regulation of cytokine production	1.0E-03		
CC	**Enrichment Score: 2.17**			
	positive regulation of smooth muscle	6.5E-05		
	GnRH signaling pathway	7.9E-01		
T	**Enrichment Score: 2.12**			
	positive regulation of protein kinase	1.7E-03		
	regulation of I-kappaB kinase/NF-ka	1.2E-02		
IM	**Enrichment Score: 2.05**			
	Interleukin receptor	1.6E-03		
	cytokine receptor activity	9.0E-03		
R	**Enrichment Score: 2.03**			
	response to vitamin	4.1E-03		
	response to lipopolysaccharide	3.2E-02		
S	**Enrichment Score: 2.02**			
	negative regulation of hormone secr	2.8E-03		
	negative regulation of transport	4.1E-03		
